# Application of Experimental Design Methodologies in the Enantioseparation of Pharmaceuticals by Capillary Electrophoresis: A Review

**DOI:** 10.3390/molecules26154681

**Published:** 2021-08-02

**Authors:** Gabriel Hancu, Serena Orlandini, Lajos Attila Papp, Adriana Modroiu, Roberto Gotti, Sandra Furlanetto

**Affiliations:** 1 Department of Pharmaceutical and Therapeutic Chemistry, Faculty of Pharmacy, University of Medicine, Pharmacy, Science and Technology “George Emil Palade”, 540142 Târgu Mureș, Romania; gabriel.hancu@umfst.ro (G.H.); adriana.modroiu@umfst.ro (A.M.); 2 Department of Chemistry “U. Schiff”, University of Florence, 50019 Florence, Italy; serena.orlandini@unifi.it (S.O.); sandra.furlanetto@unifi.it (S.F.); 3 Department of Pharmacy and Biotechnology, University of Bologna, 40126 Bologna, Italy; roberto.gotti@unibo.it

**Keywords:** chiral separation, capillary electrophoresis, experimental design, screening, optimization, quality by design

## Abstract

Chirality is one of the major issues in pharmaceutical research and industry. Capillary electrophoresis (CE) is an interesting alternative to the more frequently used chromatographic techniques in the enantioseparation of pharmaceuticals, and is used for the determination of enantiomeric ratio, enantiomeric purity, and in pharmacokinetic studies. Traditionally, optimization of CE methods is performed using a univariate one factor at a time (OFAT) approach; however, this strategy does not allow for the evaluation of interactions between experimental factors, which may result in ineffective method development and optimization. In the last two decades, Design of Experiments (DoE) has been frequently employed to better understand the multidimensional effects and interactions of the input factors on the output responses of analytical CE methods. DoE can be divided into two types: screening and optimization designs. Furthermore, using Quality by Design (QbD) methodology to develop CE-based enantioselective techniques is becoming increasingly popular. The review presents the current use of DoE methodologies in CE-based enantioresolution method development and provides an overview of DoE applications in the optimization and validation of CE enantioselective procedures in the last 25 years. Moreover, a critical perspective on how different DoE strategies can aid in the optimization of enantioseparation procedures is presented.

## 1. Introduction

Chirality is an important issue in modern pharmaceutical research, as strict conditions are stipulated by the regulatory authorities for the introduction in therapy of new chiral drugs. Food and Drug Administration (FDA) and European Medicines Agency (EMA) require the pharmacokinetic and pharmacological characterization of both racemic mixture and pure enantiomers of a drug candidate [1].

It is known that the desired therapeutic effect of a racemic substance is usually restricted to one of the enantiomers, named eutomer, while the other enantiomer, named distomer is less potent, or occasionally can present a different pharmacological effect; in some cases, it can even be responsible for the adverse effects signaled after racemic administration. In the last 25 years, the number of drugs introduced in therapy as pure enantiomers has by far exceeded the number of drugs used as racemates, underlying the importance of chirality in pharmaceutical research [1,2].

Taking into consideration the aspects mentioned above, the development of new analytical techniques for the enantioseparation of pharmaceuticals has become a necessity and a challenge for the researchers. In particular, it is of great importance to develop reliable chiral separation methods for the determination of enantiomers in different matrices, such as bulk substances, pharmaceutical preparations, and biological samples. High performance liquid chromatography (HPLC) is the most frequently used technique for the analysis of pharmaceuticals, however capillary electrophoresis (CE) is being considered today as an alternative and a complementary orthogonal approach [3].

CE offers a series of advantages in chiral separation of drugs at analytical scale, related to the rapid method development, high separation efficiency, relatively short analysis time, low consumption of analytes, reagents, and chiral selectors (CSs), high flexibility in choosing and changing CSs. Moreover, by comparison with HPLC, the use of organic solvents is much lower, consequently CE can be considered a “greener” option [4,5]. A general advantage of CE is the rapid method development, because the experimental conditions can be easily and rapidly changed from one experiment to another, without the need of changing column as it happens for chromatographic methods. In CE, usually a direct chiral separation method is used, simply consisting in the addition of CS to the background electrolyte (BGE). A relatively large number of CSs can be applied in CE, including crown ethers, macrocyclic antibiotics (aminoglycosides, glycopeptides), chiral ionic liquids, chiral surfactants; however, by far the most efficient and frequently used are the cyclodextrin (CD) derivatives (native and derivatized; neutral and ionized) [6,7].

CE is an official method of analysis in the 10th edition of the European Pharmacopoeia (Eur.Ph 10), being subject to ICH-Q4B process; however, it is official only as a general chapter, as there are no individual CE methods officinal for the chiral analysis of a certain pharmaceutical [8].

Traditionally, the development of separation methods addressed at finding the best conditions for analysis is performed by means of the univariate approach (“one factor at a time”—OFAT) where an experimental factor is varied, while the others are kept constant. Even if this approach can be efficient sometimes, it requires a relatively large number of experiments, and does not allow for the evaluation of interactions between factors, which may result in an inadequate conduction of method development and optimization, possibly leading to a false optimum [9].

Design of Experiments (DoE) may be able to overcome these limitations by producing higher quality of information and better results with a fewer number of experiments, thus proving to be a powerful tool for development of CE-based enantioresolution methods. To provide a better understanding of DoE applications, experimental designs can be divided into two groups: screening and optimization designs. To emphasize even more the actuality of the subject we can also mention that according to the recently introduced Quality by Design (QbD) concepts, analytical methods should be developed and optimized using DoE [10,11].

One of the first reviews dealing with DoE in CE, was published by Siouffi and Phan-Tan-Luu in 2000 and presents optimization strategies using chemometric approaches in chromatography and CE [12]. Sentellas and Saurina published two connected reviews regarding chemometrics applications in CE; the first part describing optimization methods while the second part dealing with data analysis [13,14]. A critical review on the development in the use of chemometric DoE based optimization methods in CE was published by Hanrahan et al. in 2008 [15].

Orlandini et al. published in 2014 an overview of applications of DoE for electromigration method optimization, including the critical discussion on the characteristics of the chemometric methods (type of design, factors, responses) used to meet a range of analytical issues, along with some practical advice and a list of the most typical challenges encountered when setting up multivariate CE approaches [16].

To our knowledge, no comprehensive review dealing exclusively with the application of DoE in the development of enantioseparation techniques by CE was published so far.

The present paper provides an updated theoretical and practical overview for the implementation of DoE in the development and optimization of CE methods used in the enantioseparation of pharmaceuticals.

## 2. Experimental Designs Applied in CE Chiral Separations

### 2.1. Selection of Experimental Design

The main purpose of method set up in analytical chemistry is discovering the conditions for an analytical procedure that produce the best possible analytical performance; in this context, DoE plays a major role [17,18]. Method set up can be divided into two phases, which represent two kinds of analytical issues: the screening and the optimization steps. In the screening step, many factors are investigated in order to identify those exerting a significant effect on the selected responses, while in the optimization step the factors are further in-depth examined for identifying the best analytical conditions for optimizing the responses [19]. After the optimization, DoE is also used in method validation for performing robustness testing and thus for evaluating the effect of small and deliberate changes in the operating conditions on the method performances, and its use is also encouraged for evaluating intermediate precision [20].

The selection of the proper experimental design to be applied should be carried out by carefully taking into consideration several aspects, mainly related to the requirements/objectives of the study and to the phase of method development. Other fundamental aspects to be considered for the choice of the right DoE are the known information, the type of polynomial model that is estimated (linear or quadratic, with or without interactions), the number of the factors, the operating cost, and time restrictions [18,21].

In general, in the screening phase and robustness testing, screening designs are applied, while during the optimization phase response surface designs are used. A screening design allows the evaluation of the effects of a relatively large number of factors from a relatively small number of experiments, so that it is possible to establish the key factors influencing the analytical performances of the method. A response surface design investigates the most important factors, generally selected on the basis of the results of the screening phase, in order to determine optimal experimental conditions by means of response surfaces or contour plots [22,23].

### 2.2. Screening Designs

Many factors, both instrumental and related to the BGE, can influence the electrophoretic enantioseparation and in general the analysis performance, it is thus necessary to evaluate which of them have a significant effect on the responses. As a matter of fact, in the first phase of CE method development the most influential factors, their optimal ranges and their interactions are not yet known, and preliminary experiments are needed [15]. The screening study is the exploration of the factors which could have an influence on the enantioseparation and is performed carrying out a minimum number of experiments on a maximum number of factors. Due to the high number of factors studied, generally, a linear model is hypothesized, and a highly fractionated design is needed. Mostly, screening design involves variations of *k* factors at *L* = 2 levels, in a relatively small number *N* of experiments *(N* ≥ *k* + 1*)* [24,25]. In addition to univariate preliminary experiments, the experimental domain is usually chosen from literature information and by means of the previous knowledge on the analytical issue under study.

The considered factors can be quantitative, i.e., changing on a continuous scale (concentration of CS, BGE concentration, BGE pH, temperature, voltage …) or qualitative, i.e., changing on a discrete scale (type of BGE, type of organic additive, type of CS …). After running the screening experiments, the evaluation of the significance of factors is performed by statistical/and or graphical analysis of the coefficients in order to highlight which factors have a significant influence on the responses. The statistical evaluation generally includes the application of t-test or analysis of variance (ANOVA). Graphical interpretation can be performed employing normal probability plots, half-normal probability plots, or Pareto charts. In the first two approaches the significant effects do not follow the normal distribution and thus will deviate from the straight line drawn by the insignificant factors while in the latter case, the standardized effects are represented as bars, and those exceeding a line corresponding to the critical t-value are considered significant [23]. The outcomes of the screening studies include the following: (i) the factors whose effect is not significant can be fixed, thus reducing the number of variables to be considered in the subsequent optimization phase; (ii) some factors which are significant can be fixed, if there is no doubt on their optimal value, or they can be further studied in the optimization phase; (iii) the experimental domain of the factors can be adjusted for approaching the analytical target, moving towards the zone leading to promising results; (iv) the optimum level for qualitative factors is established. Screening designs are also useful for validation studies, typically in robustness testing; in this case, the experimental domain is much narrower with respect to the typical ranges considered in screening studies prior to optimization [20,21].

#### 2.2.1. Two-Level Full Factorial Design

Full factorial designs (FFD) are made by experiments with every combination of factors’ levels, so that they consist in *L^k^* combinations of *L* levels of *k* factors. In two-level FFD, each factor is evaluated at two levels, called “high” and “low”, expressed as (+1) and (−1) in coded variables [22]. When *k* is small, two-level FFD can be applied for screening purposes, and *N* = 2*^k^* experiments have to be performed, allowing the estimation of main and interaction effects [26]. Anyway, FFD quickly becomes infeasible as the number of factors *k* increases, because this design is able to provide information on all interactions up to *k*-th order and thus the number of required experiments rapidly grows due to the high number of interaction terms of the model. This degree of knowledge is often unnecessary; the third and higher-order effects can often be assumed to be negligible and usually bring no useful information [24,27].

#### 2.2.2. Two-Level Fractional Factorial Design

The two-level fractional factorial design (FrFD) permits to investigate a large number of factors with a smaller number of experiments. It consists of a specific subset of experiments obtained by fractioning a 2*^k^* FFD in a 2*^k−m^* design, where *m* is the size of the fraction and 1/2*^m^* is the fraction of the original FFD [23]. The design must be balanced and chosen so that the experiments map the experimental domain as well as possible, and orthogonality is preserved [24]. Different FrFD can be created for a large number of factors; the size of the fraction influences the number of effects that can be estimated, and the number of experiments needed [24,28]. A drawback of fractionating is that some information is lost because only certain coefficients of the model are clearly calculated. As a matter of fact, FrFD does not enable the estimation of all main and interaction effects separately, because some of them are confounded, namely, they are estimated together [21,25,29].

##### Plackett-Burman Design

In Plackett-Burman design (PBD) *L* = 2 levels (−1, +1) are considered for each factor. This matrix makes it possible to estimate linear models without interactions and thus it finds useful application also in robustness testing. Anyway, in most cases, it can be assumed that the influence of interactions of third-order or higher is negligible and thus they can be excluded from the polynomial model [28]. The greatest advantage of PBD consists in the possibility of screening a high number of factors with a low number of experiments: a maximum of *k* = *N* − 1 factors can be examined in *N* experiments, where *N* is a multiple of four [25]. As an example, this means that only 8 experiments are needed for studying the effects of 7 factors, while only 12 experiments are required for studying the effects of 11 factors. PBD is cyclical, the matrix is built from starting sequences with coded factor levels for the first row (first experiment). The remaining rows of the matrix are generated by cycling the end code to the beginning of the row and moving the rest of the codes one place to the right. The sequence ends with all (−1) values, which corresponds to the last experiment (last row) in the matrix [24,25,27]. When *k* is higher than the number of real factors to be examined, the remaining columns of the PBD are defined as dummy factor columns, for which the change between the levels (−1) and (+1) has no physicochemical meaning [24].

#### 2.2.3. Asymmetric and Symmetric Screening Designs

Asymmetric screening designs (ASD) and symmetric screening designs (SSD) show a great usefulness in gaining preliminary information on the influence of the variables under study. The number of levels *L* for each factor is the same in symmetric designs and is different in asymmetric designs; in any case *L* ≥ 2 (usually from 2 to 4). The possibility to evaluate the effect of more levels of the same factor is an interesting characteristic, because even if these designs cannot give a response surface as a result, they can furnish a clear indication of the trend of the response obtained at the different levels of the factors. In this way, it is possible to obtain valuable information for approaching the optimum values for the factors and for giving indications on the choice of the experimental domain to be studied in the subsequent optimization phase [26].

### 2.3. Optimization Designs

The optimization designs permit to obtain experimental data which can be fitted in a quadratic polynomial model for generating a response surface, which describes the behavior of the data set and makes previsions all throughout the experimental domain [17]. Response surface methodology (RSM) refers to multivariate techniques that can generate response surfaces and provide solutions for selecting the optimum conditions, so that the response is maximized, minimized, or even targeted to an optimal value [22,30]. In RSM phase, first-order polynomial models with interactions, and consequently designs as FFD, can be used only when the data do not present curvature, but usually, it is necessary to use experimental designs capable to estimate the coefficients of second-order polynomial models [17]. This is possible by employing designs where for each factor *L* ≥ 3, because two levels designs cannot give information about maxima or non-linear relationships. Another important aspect is the efficiency *E* of the designs, determined as *E = p/f*, where *p* is the number of coefficients in the polynomial model to be fitted and *f* is the number of factor combinations in the experimental design [29]. As a matter of fact, when hypothesizing a quadratic polynomial model, the number of coefficients to be estimated rapidly increases with the increase of the number of factors to be evaluated. For this reason, for carrying out RSM it is often desirable at a first stage to reduce the number of factors via screening designs as described above. In this way, it is possible to select a small number of main factors (three-five) to be studied in detail, for which both squared, and interaction terms are of interest [31].

In RSM, qualitative factors cannot be examined; only quantitative and mixture-related factors can be included in the study. Moreover, in order to be able in calculating models for making previsions, it is necessary to perform some replicates of the center point or duplicates/triplicates of each run. From these replicates, it is possible to obtain an estimation of experimental variance, by which the validity of the model can be established. After the statistical evaluation of the model has been performed, two-dimensional contour plots or three-dimensional response surfaces can be drawn to visualize the trend of the predicted response [22,23].

When selecting a response surface design, some of the desired features include: (i) reasonable distribution of data points in the region of interest; (ii) allowing model adequacy, including lack of fit, to be investigated; (iii) not requiring a large number of runs; (iv) not requiring too many levels of the factors. Moreover, it is important for the second-order model to provide good predictions throughout the region of interest; thus, it has been suggested that the design should be rotatable, namely the variance of the predicted values is uniform at all points that are at the same distance from the design center. These desired features can be sometimes conflicting, thus correct judgments by the researcher must often be applied for selecting a suitable design [22].

Three-level full factorial design

Three-level FFD is seldom used, because the number of experiments is quite high (*N* = 3*^k^*). It contains all possible combinations between the *k* factors and their *L* = 3 levels, and the efficiency is rapidly lost with the increase of the number of the factors to be studied [17,21].

Orthogonal array design

Orthogonal array design (OAD) is a kind of FrFD, based on a design matrix with selected combinations of multiple factors at multiple levels. According to the orthogonal array properties, between each pair of columns each combination of levels appears an equal number of times. By this design it is possible to estimate the main effects and two-factor interactions. OAD can be used both in the screening and in the optimization step as long as the data do not present curvature [22].

#### 2.3.1. Central Composite Design

Central composite designs (CCD) are made of two-level FFD points (*2^k^* experiments), star points (*2k* experiments), and a number of replicates *c_p_* carried out at the center point, so that the number of experiments is *N = 2^k^ + 2k + c_p_*. The factors are studied at five levels (−*α*, −1, 0, +1, +*α*). All the factors in the star points are set to 0, apart from one factor which assumes the value *±* *α*. The *α* value depends on the number of factors and is usually included between 1 and √*k*. The design is made rotatable by the choice of *α* value: *α =* (*f*)^1/4^ yielding a rotatable CCD, where *f* is the number of factorial design points [18,22]. CCD can be classified in circumscribed designs, where the star and the factorial points lie equidistant from the center and *|α|* > 1, and in inscribed designs, where the star points lie within the space of the factorial design and +*α* and −*α* are equal to the boundaries [21,24]. There is also a third kind of CCD, called Face Centered Central Composite Design (FCCD), where *α* = 1, so that the star points lie on the faces of the hypercube, namely the faces of the factorial design [24]. FCCD is often used because it requires only three levels for each factor, and this can represent a practical advantage because in some cases it can be difficult to change factor levels; anyway, it is not rotatable [22].

#### 2.3.2. Box-Behnken Design

Box-Behnken design (BBD) is a modification of a three-level factorial design, where one factor is kept at the central value in every experiment, plus at least one experiment at the center. BBD can only be implemented for three or more factors; each factor is studied at three levels, the experimental points are located on a hypersphere and are placed equidistant from the central point [17,30]. The number of experiments is *N* = 2*k* (*k* − 1) + *c_p_*, thus BBD designs are very efficient in terms of required runs, and they are either rotatable or nearly rotatable [21,22]. The highest level and lowest level combinations for every factor are not included in the design, thus this matrix is useful for avoiding experiments in the extreme conditions, especially when these points represent combinations that are prohibitively expensive or impossible to test for physical or technical constraints [22]. For the same reason, its use should be suggested for the cases where the optimum is expected to lie in the middle of the factor ranges [21].

#### 2.3.3. Doehlert Design

In Doehlert designs (DD) the experimental points are distributed on a spherical shell. The matrix is not rotatable, but it presents high efficiency, since the number of experiments required is *N* = *k*^2^ + *k* + *c_p_* [22]. As for the distribution of the experimental points, for two factors DD is a regular hexagon, while for three factors it is a geometrical solid called centered dodecahedron [17]. Each factor is studied at a different number of levels: one at three, one at five and all the other factors at seven levels. This can be useful to differentiate the study of the variables, since the factors which are considered more important, or which have a larger domain, can be tested at more levels [21]. At the same time, this could represent a drawback when a narrow experimental domain cannot be practically and easily divided into seven levels. Another characteristic of DD is that the levels are uniformly distributed, so there are equal distances between all neighboring experiments. Moreover, the most important advantage is the potential for sequentiality: according to the obtained results, it is possible to move the experimental matrix towards another experimental domain for approaching the optimal zone, using previously tested points from the first matrix [24].

#### 2.3.4. D-Optimal Designs

Optimal designs are generated based on computer algorithms and can be used both for screening and optimization. They are especially useful when the desired number of runs is smaller than those required by a classical design or when the experimental regions are irregular [19,24,30]. The experimental domain is represented by a number of candidate experiments and some mathematical criteria are used for choosing good sets of candidate points. Among the different criteria available for the selection (D-criterion, G-efficiency criterion, A-criterion, I-criterion, and others), the most common is D-optimality [19,30,32,33]. The experiments of the D-optimal design (D-oD) are selected as the combination of experiments with the maximum determinant for *X^T^X*, where *X^T^* is the transpose of the model matrix *X*. It can be demonstrated that the precision of the coefficients estimate is directly correlated with the *X^T^X* matrix. This leads to the minimization of the confidence interval for each coefficient, and thus to a higher precision of the estimate [24,25,26]. An important characteristic of D-oD is that the researcher can specify the maximum number of experiments that can be performed and can choose among different matrices of different quality, made by a different number of experiments. The minimum number of runs depends on the number of coefficients of the model, while the maximum number is chosen by the researcher depending on the constraints imposed by the cost of experiments, the time available, the desired quality of information. In this way a good compromise between the quality of the matrix and the experimental effort can be achieved [19].

### 2.4. Model Evaluation and Validation

Once the polynomial model has been calculated, several tools are available for statistically evaluating its quality. The ANOVA allows to verify the model significance, namely whether the factors can explain a significant amount of the variance in the response variable. Moreover, if replicates have been performed or if an estimate of the pure error is available, the validity of the model can also be checked [24]. ANOVA divides the total variation of a selected response ($SS_{tot}$, total sum of squares) into a part due to the regression model ($SS_{regr}$, regression sum of squares) and a part due to the residuals ($SS_{res}$, residual sum of squares):$\;SS_{tot}=SS_{regr}+SS_{res}$. If replicates are made, the residual sum of squares can be further partitioned into the sum of squares due to pure error ($SS_{pe}$) and due to lack of fit ($SS_{lof}$): $SS_{res}=SS_{pe}+SS_{lof}$. By means of comparing the related mean squares *MS*, obtained by dividing the *SS* values by the associated degrees of freedom, it is possible to evaluate the model significance and the model validity by means of a one-sided *F*-test [19,24]. In order to test the significance of the regression, the main squares due to regression $MS_{regr}$ is compared with the residual mean square $MS_{res}$. If $MS_{regr}\;$is significantly larger, it means that the model is significant. Instead, the lack of fit test to check for validity is performed by comparing $MS_{lof}$ to $MS_{pe}$. If $MS_{lof}\;$is significantly larger than $MS_{pe}\;$the model is inadequate, since the distance between measured and predicted values of the response cannot be explained in terms of the pure experimental uncertainty; if $MS_{lof}$ and $MS_{pe}\;$are comparable, the model is justified [24]. Sometimes the lack of fit might be so small that it appears of no practical importance, but the *F*-test for lack of fit will show that it is statistically significant. This often occurs when the model fits well the data, but the experimental variance is very low. In this case, from the practical point of view, if the residuals are small enough to be considered acceptable, validity doesn’t need to be verified [29].

Residual plots, where the residuals are plotted against the predicted values of the response, can be also used for the evaluation of the model. The model is adequate when no particular trend in the pattern of the residuals is observed and they are randomly scattered, with a number of positive residuals approximately equal to the number of negative residuals. This tool is particularly useful to verify if a linear model is inadequate to describe the data; in this case, higher residuals (U-shaped) will be found at the center of the plot, showing that there is a lack of fit with the linear model and that the data are better represented by a curve [24].

Other parameters that are recommended to be used in evaluating the fit of a model are the value of the coefficient of determination *R*^2^, adjusted coefficient of determination *R*^2^*adj*, and predicted variation *Q*^2^. The coefficient of multiple determination is calculated as $R^{2}=SS_{regr}/SS_{tot}=1-SS_{res}/SS_{tot}$ and is used to estimate the proportion of variation explained by the regression, ranging from 0 to 1. However, its value increases with the number of terms included in the model, either if a variable is significant or not. Therefore, it is advantageous to use the adjusted coefficient of determination $R_{adj}^{2}=1-\frac{n-1}{n-p}*\left(1-R^{2}\right)$, which accounts for the number of model parameters *p* determined based on *n* number of experiments and has an optimum when additional terms do not provide further value to the model [34]. The coefficient of predicted variation *Q*^2^ is the fraction of the total variation of the response that can be predicted in the model and it is calculated as $Q^{2}=SS_{tot}-PRESS/SS_{tot}$, where *PRESS* is the Predicted residual error sum of squares, which is calculated by leave-one-out cross-validation. This represents a measure of how well the model will predict the responses for new experimental conditions [19,22,24].

After a general evaluation of the proposed model, it may occur that the implementation of data transformation allows a better fit. Mathematical transformations apply a mathematical function to all the measured responses, generating a new set of data, and then a new model can be built to better explain the data behavior. Moreover, before moving on with a model, it is advisable to attempt model refinement. This can be done in a sequential way, through the exclusion of the factors that are found to be not significant in the coefficient plot, to obtain an improvement in the model predictivity *Q*^2^ [19,28].

Finally, if the model has been built for prediction purposes such as in RSM, it is particularly important to extend the validation to independent data. Thus, new experiments should be performed, and the measured observations will be compared with the predictions from the model in order to verify their agreement [24].

### 2.5. Application of DoE in Analytical QbD

The application of DoE in the development of separation methods undoubtedly gives important advantages, making it possible to obtain a high quality of information with a limited number of experiments. Anyway, it has to be highlighted that further improvements in knowledge can be attained by applying QbD principles. The concept of QbD has been introduced by the FDA as a quality paradigm to be used in the pharmaceutical field, with the aim of explicitly designing quality into pharmaceutical products and processes. QbD was approved by International Council for Harmonization (ICH), and dedicated ICH guidelines were published shortly after [35,36,37,38]. This approach is based on process understanding and control by quality risk management and has been transferred to the development of analytical methods, with the name of Analytical Quality by Design (AQbD) [39,40,41,42,43,44]. Following the AQbD workflow, the method development can be divided into major several steps: (i) definition of analytical target profile and method scouting; (ii) risk assessment and definition of critical method parameters (CMPs) and critical method attributes (CMAs); (iii) DoE for evaluating the effects of the CMPs on the CMAs; (iv) definition of the design space (DS) or method operable design region (MODR); (v) method control. By means of this systematic development it is possible not only to find the optimal conditions of analysis, but also to obtain robust analytical methods. Within this framework, a key role is exerted by DoE, which gives the possibility of calculating mathematical models relating the CMPs to the CMAs and locating the optimum zone by using multicriteria decision-making tools. In general, at first screening designs are performed focusing on all the CMPs selected by risk analysis, and then the optimization phase by RSM is carried out to gain in-depth knowledge on the effect of selected CMPs.

### 2.6. Analytical Design Space and Method Operable Design Region

When a number of responses have to be optimized simultaneously by DoE, some multicriteria decision-making tools are available, such as desirability studies or response overlay. Individual response values can be combined in an objective function in order to achieve an optimum value for all the responses [45], or the response surfaces can be overlaid in order to graphically evidence the sweet spot, i.e., the zone where all the requirements are fulfilled [18]. Anyway, it has been established that when developing an analytical method by AQbD, for defining the optimum zone it is necessary to consider not only the predicted values for CMAs, but also the probability that the CMAs requirements are fulfilled; in other words, the definition of the DS is the main target [41,43].

In ICH Q8, the DS has been defined as “the multidimensional combination and interaction of input variables (e.g., material attributes) and process parameters that have been demonstrated to provide assurance of quality” [35]. For analytical methods, this has been identified as a multidimensional space that includes any combination of the variables that have been demonstrated to provide assurance of quality of the data produced by the method [38,39]. In particular, the MODR for analytical methods has been defined as the equivalent of the DS for manufacturing processes [43,44]. This is identified as the zone where the requirements for the CMAs are fulfilled with a certain probability and a selected risk of error, and it is computed by considering the uncertainty of the model parameters [43]. For achieving this aim, different methods such as Bayesian modelling, bootstrapping techniques or Monte-Carlo simulations can be used [43]. Among these, Monte-Carlo simulations combined with interpretation of DoE response modelling make it possible to estimate method uncertainty and thus to provide an adequate level of probability that the CMAs specifications are met. Once the MODR has been calculated, the working point to be used for routine analysis can be selected anywhere inside the MODR. From the regulatory point of view, this means that a more flexible approach is achieved, because any movement within the MODR is not considered a change, hence no revalidation of the method is required [35,43].

## 3. Application of DoE in the Development of Chiral CE Methods

A relatively large number of articles dealing with applications of experimental design approaches in the development of CE techniques (capillary zone electrophoresis—CZE, micellar electrokinetic chromatography—MEKC, capillary electrochromatography—CEC) for the analysis of chiral pharmaceuticals has been published. The following part of the manuscript is a summary of the most important studies arranged chronologically in the frame of each of the CE separation modality; the main aspects and trends in the application of experimental design in chiral CE during the last 25 years are presented and discussed.

The articles were selected based on a literature screening survey on Google Scholar, Pubmed, Scopus and Web of Science databases; the keywords used in the survey were: chiral separation, enantioseparation, enantiomeric purity control, capillary electrophoresis, experimental design, and quality by design.

### 3.1. Capillary Zone Electrophoresis (CZE)

CZE is the simplest capillary electrokinetic separation mode, based on the differences in the charge-to-mass ratio of the analytes, hence all factors affecting the charge of the analytes play an important role in selectivity adjustment. In particular, BGE pH, nature and concentration, CS type and concentration, can influence the migration velocity of the analytes and separation. Supplementing the BGE with CDs, is undoubtedly the most popular and successful approach to gain direct enantioresolution in CE. Under these condition, transient diastereomeric host-guest type complexes with different inclusion constants can be easily formed between the CD cavity and the two enantiomers, thus differentiating their mobility. Noteworthy, in CE the separation can also be accomplished in the case of enantiomers possessing the same binding constants with the CD, due to the different mobility of the diastereomeric complexes [4]. Since inclusion complexation occurs via Van der Waals, hydrophobic and electrostatic interactions as well as hydrogen bonding, the enantioseparation is strongly affected not only by the nature of the CD but also by all of the conditions influencing the inclusion complexation [4,6,7]. Thus, optimizing a chiral separation involves the selection of several variables making the DoE the ideal approach facing the challenge.

One of the first articles where DoE was applied for the development of a chiral separation method by CE was published in 1994 by Rogan et al. for the enantioseparation of clenbuterol, a sympathomimetic β-amino alcohol used in therapy as bronchodilator. A PBD was applied to study simultaneously the effects of BGE concentration, BGE pH, β-CD concentration, organic modifier (methanol) concentration, and injection time on chiral resolution, migration time and peak efficiency. BGE concentration, BGE pH and CD concentration were found to have significant effects on chiral resolution; migration time was strongly influenced by BGE pH, while BGE concentration, BGE pH and injection time affected peak efficiency. The results were compared with those obtained in a study where an OFAT approach was used in the optimization process, published previously by the same research group, in which good chiral separation was obtained using β-CD as CS in a citrate/phosphate BGE at pH 4.0 [46]. Similar information but with much fewer experiments was obtained in the case of PBD in comparison with OFAT. The CE method was compared with a HPLC method using CDs as CSs, to identify a rational basis for the transfer of knowledge on optimization between HPLC and CE. A generic optimization strategy for the enantioseparation of new compounds, for which no previous investigations have been carried out before was also proposed [47]. It was stated probably for the first time that in chiral CE, scouting experiments have to be performed before an experimental design-based optimization to acquire an indication of the chiral selectivity, peak shape, and analysis time.

A CCD was applied by Small et al. for the development of a chiral separation method by CE for amlodipine, an asymmetric dihydropyridine type calcium channel blocker. α-CD was used as CS in an acidic phosphate BGE at pH 3.16. After an initial OFAT investigation on the influence of several analytical parameters on chiral resolution, three factors (BGE pH, α-CD concentration, temperature) were selected as significant and used in a CCD, while the others (BGE concentration, voltage) were kept constant during the optimization process; chiral resolution and Kaiser peak separation function were used as responses. Optimum values predicted by the application of CCD were in excellent agreement with those observed experimentally using the optimized parameters [48].

Boonkerd et al. investigated the enantiomeric purity of dexfenfluramine, a serotoninergic anorectic drug by CE using dimethyl-β-CD (DM-β-CD) as CS. Two PBDs were applied in the optimization process; the influence of BGE pH, DM-β-CD concentration, methanol concentration, temperature and voltage on the chiral resolution, migration time and the tailing factor of the last eluting peak was investigated. Interestingly, the first two responses were combined in a composite quality response, to make a compromise between them. The experimental conditions described previously by Bechet et al. were used as a starting point of the method development and compared with the lower levels and higher levels in the two complementary designs [49]. The reason for performing two parallel designs involving the same factors and responses may have the purpose to cover a larger experimental domain. The optimized method was applied for the determination of levofenfluramine as chiral impurity in dexfenfluramine samples, however, the resolution values below 1.5 may not be enough for the quantitative determination of the second migrating distomer [50].

Varesio et al. developed a CE chiral separation method for the enantioseparation of five amphetamines (amphetamine, methamphetamine, 3,4-methylenedioxyamphetamine (MDA), 3,4-methylenedioxymethamphetamine (MDMA), 3,4-methylenedioxyethylamphetamine (MDEA)). A CCD was used for method optimization, the influence of BGE concentration, BGE pH, CD concentration, temperature and voltage on chiral resolution, migration time and generated power was investigated. The results were compared with the ones described previously in a OFAT method. The use of experimental design approach allowed the separation to be developed using fewer experiments than a univariate development would have needed. Also, response surfaces were drawn making it possible to visualize the robustness of the separation [51].

Fanali et al. developed a chiral separation method by CE for the enantioseparation of epinephrine. A neutral CD, heptakis-2,6-di-O-methyl-β-CD (HDM-β-CD), and an ionized CD, carboxymethyl-β-CD (CM-β-CD), were used as CSs in a pH range between 2.5 and 7.0; CM-β-CD gave the best results and was selected for the subsequent optimization. A FrFD with five variables was used for screening purposes and a CCD with three selected variables from the previous design was used for optimization in the method development. Chiral resolution and migration times were selected as responses, a desirability function was used to select a solution that assures minimization of migration times and maximization of chiral resolution. The response surfaces obtained were used to establish the zones of optimum robustness [52].

Guillaume and Peyrin proposed a chemometric method for the optimization of a chiral separation method by CE of a mixture of 4 imidazole derivatives (bifonazole, econazole, miconazole, sulconazole). The optimization process was composed of 9 or 18 preliminary experiments, based on a FrFD, followed by further optimization by simplex methodology. The selected factors were BGE pH, acetonitrile concentration in the BGE, β-CD concentration and temperature, while the responses were chiral resolution, migration time and number of theoretical plates. A phosphate BGE at pH 4.70, acetonitrile as BGE additive and β-CD as CS were used in the optimized separation [53].

β-blockers are among the most interesting pharmaceuticals from stereochemistry point of view, as all compounds are chiral and different activity between enantiomers has been clearly established. Vargas et al. developed a generic chiral separation method applicable in the case of 11 β-blockers (acebutolol, alprenolol, atenolol, bunitrolol, labetalol, metoprolol, oxprenolol, pindolol, propranolol, sotalol, toliprolol). An acidic phosphate/triethanolamine BGE and neutral DM-β-CD, hydroxypropyl-β-CD (HP-β-CD), and ionized CM-β-CD, sulfobutylether-β-CD (SBE-β-CD) as CSs were used. Ionized CDs proved to be efficient in the separation of basic β-blockers; CM-β-CD in eight cases while the other three were enantioresolved using SBE-β-CD. Two different FrFDs were used, one examining four factors at three levels (3^4-2^) and the other examining three factors at two levels (2^3-1^). The selected factors were the type of CD, CD concentration, BGE pH and percentage of methanol in the BGE; the selected responses were chiral resolution and analysis time. The three-level FrFD was used to select the optimum CD, while the two-level design was used to improve enantioresolution. The same strategy is suggested to be efficient in the separation of other groups of pharmaceuticals [54]. The study proves applicability and utility of DoE in the development of generic chiral separation methods used in the enantioseparation of several pharmaceuticals with similar physicochemical and structural characteristics.

The enantioseparation by CE of the β-blocker celiprolol, was reported by Daali et al. using sulfated-β-CD (S-β-CD) as CS, in polyvinyl alcohol coated capillaries. An initial evaluation of several CDs at two different pH levels was performed, and the best results were obtained using acetate BGE at pH 4.0 and S-β-CD as CS. A CCD was applied for optimization, considering as the selected factors the BGE concentration, BGE pH, S-β-CD concentration, and temperature; the selected responses were chiral resolution, analysis time and generated current. By studying the response surface of the quadratic model, the optimal region which provides method robustness was established. The method was validated and used for the determination of celiprolol enantiomers in tablets and urine samples [55].

de Boer et al. developed a CE method for the chiral separation of seven β_2_-sympathicomimetic drugs (clenbuterol, fenoterol, isoprenaline, oxedrine, salbutamol, ritodrine, terbutaline) using HP-β-CD as CSs in a polyethylene glycol (PEG) solution. Correlation between the chemical structures of the analytes and CD were established; the compounds having two hydroxylic groups substituted at the aromatic ring (isoprenaline, fenoterol, terbutaline) were more easily separated, because their geometric structure better fits in the β-CD cavity. A two factor CCD was used in the optimization process, using PEG and HP-β-CD concentrations as variables and chiral resolution as response. The target for chiral resolution was set to a minimum of 2.5, in order to use the developed method for chiral impurity control (concentration as low as 0.1%) [56].

Zhu and Lin applied orthogonal and uniform designs for the optimization of chiral separation of four model substances (disopyramide, ketamine, norfenefrine, synephrine) using DM-β-CD as CS. The BGE pH, DM-β-CD concentration, voltage, and temperature were set as variables in the design while chiral resolution was measured as response. Orthogonal designs provide a quick and efficient method for identifying the significance of individual parameters and establishing the optimum analytical conditions; however, uniform designs are a more efficient tool when dealing with a high number of factors and levels [57]. Combining uniform and orthogonal design for a complex separation system could be a smart option in chiral separation method development, however, the presented approach does not allow the study of second order and interaction effects, which can play a crucial role in CE chiral separations.

Perrin et al. used a DoE strategy for the chiral separation of 14 amino acids by CE. Three CDs were used as CSs: HP-β-CD, CM-β-CD and SBE-β-CD. The examined factors were CD type, CD concentration, BGE pH and organic additive (methanol) concentration in the BGE; chiral resolution was used as the response. Two types of FrFDs were applied depending on the analytes: a 3^4-2^ FrFD (4 factors at 3 levels) and a 2^3-1^ FrFD (3 factors at 2 levels). Among the 14 amino acids, 12 were enantiomerically baseline separated, while 2 amino acids were not separated, and other separation systems were necessary. A generic separation scheme was developed for the chiral separation of amino acids by DoE approach; however, no generalization of the optimum analysis conditions was possible within this group of compounds [58].

In another study, Gotti et al. used PBD for screening of factors and robustness testing of a CE method for the chiral separation of salbutamol using a glycosaminoglycan, dermatan sulphate, as CS. RSM using a DD was applied after the preliminary screening design; CS concentration, BGE pH and voltage were selected as parameters and chiral resolution and analysis time were chosen as the responses. Derringer desirability function was used to simultaneously optimize the two responses. The method was applied for the determination of salbutamol enantiomers in spiked human urine samples [59]. The study shows the applicability of experimental design in CE chiral separation when other CSs than CDs are used in the separation.

A chiral CE method using CDs as CS was developed by Mateus et al. for the enantioseparation of atropine, a tropane structure alkaloid found in plants from Solanaceae family. Prior to optimization, a complex CD screening was performed to establish the optimum CS, and the best results were obtained with S-β-CD. A CCD was used for the characterization of BGE concentration, BGE pH and S-β-CD concentration effects on the chiral resolution, migration time and generated current. Under these optimized conditions, baseline separation of littorine and atropine enantiomers was achieved in less than 5 min, with high resolution values. The method was applied for the enantioseparation of atropine in a pharmaceutical preparation (ophthalmic solution) and for the enantiomeric purity evaluation of (−)-hyoscyamine in plant extracts, which can be useful in selecting the best extraction procedure. Interestingly, the results showed that supercritical fluid extraction induced less racemization than classical liquid-solid extraction procedures [60].

Loukas et al. developed and validated a chiral separation method for peptides using CE, CD as CSs and applying DoE for development. Ala-PheOMe was used as model peptide while HP-β-CD as CS. A FCCD was applied; the selected factors were BGE concentration, BGE pH, CD concentration and voltage while the responses were chiral resolution and migration time. To optimize the two analytical responses (maximize resolution, minimize migration time) Derringer desirability functions were used [61]. In other types of designs, the same strategy may be applied to find the levels of the predictor variables that maximize overall response desirability: first, an effective prediction equation based on the levels of the factors to match the observed product characteristics is used, and then the levels of the factors that generate the most desirable predicted values simultaneously are found.

In another study, Brunnkvist et al. developed a CE method for the chiral separation of a tetrapeptide H–Tyr–(D)Ala–Phe–Phe–NH_2_. Two CSs, 18-crown-6-tetracarboxylic acid and HDM-β-CD were tested observing that the crown ether gave better results; an acidic phosphate BGE at pH 3.0 was used. An FFD was employed for robustness testing; the factors BGE concentration, BGE pH, CS concentration and temperature were varied at two levels, chiral resolution was used as response [62].

Saavedra and Barbas developed a chiral separation method by CE for lactic acid using a DoE strategy. An initial complex CD screening was used to identify the optimum CS, the best results were obtained when using HP-β-CD. Two three-level FFDs were used to optimize analytical conditions; BGE concentration, BGE pH and HP-β-CD concentration were used as variable factors, chiral resolution was used as analytical response. After the first design, pH value was fixed at the value of 6.0 and in the second design the experimental domains for both BGE concentration and HP-β-CD concentration were moved to higher values. The method was validated for the determination of lactic acid in plasma allowing the identification of both lactic acid isomers in body fluids such as urine, amniotic and cerebrospinal fluids [63].

Harang et al. developed a CE chiral separation method for the enantioseparation of propranolol using cellobiohydrolase (Cel7A) as CS. To identify significant experimental variables, a series of preliminary experiments were carried out, followed by a FCCD, where the influence of BGE pH, ionic strength, and organic additive (acetonitrile) concentration on chiral resolution and separation selectivity, were evaluated. A high acetonitrile concentration, high ionic strength and low pH resulted in improved peak symmetry and efficiency, but at the expense of resolution and selectivity [64].

A DoE strategy was used for the CEC enantioseparation of a new synthesized aryl propionic acid, namely 2-[(4′-benzoyloxy-2′-hydroxy)phenyl-propionic acid], with potential anti-inflammatory effect. Vancomycin was used as CS. Partial filling-counter current method has been used to avoid the presence of the absorbing CS in the path length of the detector and to increase sensitivity of the method. A CCD was used in the optimization, the influence of CS concentration, BGE pH and temperature on chiral resolution and migration times was studied. Derringer desirability function has been used to simultaneously optimize the two analytical responses [65].

A CE method was developed by Awadallah et al. for the CE enantioseparation of ofloxacin, a fluoroquinolone antibacterial derivative, in highly diluted samples (20–700 ng/mL for each enantiomer). Ofloxacin is used in therapy both in the form of racemate, and as pure enantiomer, levofloxacin. Methyl-β-CD (M-β-CD) was used as CS testing two injection methods: hydrodynamic and electrokinetic injection. A fluid-fluid extraction from physiological solution was applied and electrokinetic injection was employed to improve the sensitivity. A FCCD was used for method optimization, investigating the influence of BGE concentration, BGE pH, M-β-CD concentration and temperature on chiral resolution, migration time, peak area, and generated current. The limit of quantification (LOQ) was found to be 11.4 ng/mL for *S*-ofloxacin and 10.8 ng/mL for *R*-ofloxacin [66].

Marchesini et al. developed a chiral separation method for ketamine, a general anesthetic, using CM-β-CD. A screening PBD, followed by a CCD optimization. The method was applied for the determination of ketamine in pharmaceuticals [67].

Perrin et al. studied the effects of peak measurement and analysis parameters on chiral CE, using as model compound an H1 antihistaminic derivative, dimethindene. The influence of parameters related to UV detection (detection and reference wavelengths, detection, and reference wavelength bandwidths), signal processing (data acquisition rate, filter, and filter peak width), and peak detection (detection threshold, peak width) on chiral resolution, peak area, and signal-to-noise (S/N) ratio, were studied at two different concentrations levels (high and low S/N ratio) using a DoE strategy. A D-oD was first used for screening the nine factors in ten experiments to identify the most significant parameters, followed by a FCCD for modelling different responses as a function of significant parameters, namely data acquisition rate, filter, and filter peak width. The results revealed that when designing a CE separation, signal-processing parameters should be carefully chosen because setting these parameters at various levels will cause significant differences in the measured separation, the S/N ratio, and the peak area [68]. Interestingly, peak measurement/analysis parameters should be optimized as much as the method’s chemical and physical parameters, since significant improvements in impurity detection and/or identification, which is a crucial aspect in CE, can be achieved. The study also points out that the robustness of a system can be severely harmed when the parameter settings are transferred from one instrument to another.

The robustness of a generic CE chiral separation using highly sulfated CDs (HS-CDs), a low pH phosphate BGE, and the short-end injection technique was tested by Perrin et al. The robustness was evaluated for the enantiomeric separation of propranolol (basic drug), praziquantel (neutral drug) and warfarin (acidic drug). The influence of eight factors (BGE concentration, BGE pH, HS-CD concentration, temperature, voltage, sodium hydroxide rinse volume, BGE rinse volume, injection time) which were considered to significantly affect the separations, was studied by means of PBD [69]. The evaluation of chiral separations was made in the context of developing a generic CE separation strategy for different compounds. The study demonstrates the usefulness of DoE methodologies in robustness testing and hereby the importance of recognizing the experimental parameters which should be strictly controlled during the separation.

A screening strategy for the development of enantiomeric separation methods in CE was proposed by Jimidar et al., based on a screening design as a function of pH to determine the optimal separation conditions followed by a selection of the optimum CS by Taguchi designs. In this approach several variables, such as the type and concentration of CDs, BGE concentration, percentage of organic modifier in the BGE, were varied simultaneously; their effect on the chiral resolution and migration time was evaluated. The strategy was applied for the chiral separation of several chiral analytes under development [70]. In this study a general strategy for the development of enantiomeric methods for basic, neutral, and acidic compounds is proposed, which can be useful in managing resource time efficiently in development laboratories. However, it can be noticed that capillary temperature which can have a crucial effect on the chiral resolution, was not considered in the proposed strategy.

A comparative CE versus HPLC study was performed by Zhang et al. for the chiral separation of 11 phenyl alcohol derivatives using S-β-CD as the CS in CE and a Chiralcel OD-H column in HPLC. A DoE strategy was applied to determine the effect of BGE pH, S-β-CD concentration and voltage on the chiral resolution, migration time and selectivity. Moreover, the effect of the analyte structure on enantioseparation was evaluated: CD concentration, the location of the aromatic group with respect to the asymmetric center of the analytes and the substituent group connected to the chiral carbon, were found to be factors influencing the complexation, selectivity, and resolution [71]. The comparative study demonstrated that CE offers some advantages compared to HPLC for the separation of the considered analytes, related to the shorter analysis time, and lower operational cost.

Jimidar et al. applied a DoE approach for the CE chiral separation of an experimental drug (R209130) with three asymmetric carbon atoms which led to eight stereoisomers. A dual CD system consisting of α-CD and S-β-CD was selected as CS. The initial separation conditions were optimized using a BBD; BGE concentration, α-CD concentration, S-β-CD concentration, and voltage were selected as variables, chiral resolutions between enantiomers and the migration time of the last peak as the responses. The method was validated and proved to be suitable for chiral impurity control [72].

A multivariate optimization approach for CE chiral separation using human serum albumin (HSA) as CS was published by Martinez-Gomez et al. Affinity electrokinetic chromatography–partial filling technique was used, and four basic model compounds were selected (alprenolol, oxprenolol, promethazine, propranolol). A BBD was applied in the optimization process; BGE pH, HSA concentration and HSA solution plug length were selected as variables. Pareto charts and partial least squares analysis were used to examine the effects of experimental variables on chiral resolution. In addition, the effect of the physico-chemical properties of model analytes, acid–base ionization constants, octanol-water partition coefficients and analyte–HSA protein binding percent on the chiral resolution was established. The results reveal that analyte–HSA interaction strength and the consequent enantiomer resolution, is largely determined by analyte hydrophobicity; when the hydrophobicity decreases, the range of experimental conditions that yield enantioresolution, narrows [73].

An interesting study was published by Elek et al. in which two different dual CS systems containing a CD derivative (M-β-CD, DM-β-CD) and a new diaza-crown-ether derivative (N-[2-(1,4,10,13-tetraoxa-7,16-diazacyclooctadecan-7-yl)propanoyl]glycine) were studied in the chiral separation of tryptophan- and tyrosine-methylester enantiomers. A CCD was used for optimization; CD concentration, crown-ether concentration and BGE concentration were set as variable factors whereas chiral resolution and migration time were selected as responses. The dual CS systems proved to enable better chiral separation of amino acid derivatives [74].

Zhang et al. developed a CE method for the chiral separation of tamsulosin, an α1 receptor antagonist, used in the treatment of benign prostate hyperplasia in the form of pure enantiomer, *S*-tamsulosin. S-β-CD was chosen as CS. Response surface methodologies such as BBD, FCCD, and central composite circumscribed (CCC) design, were comparatively used for the optimization. CD concentration, voltage and temperature were chosen as variable factors, and their influence on chiral resolution and migration time was observed. The design points for all three approaches are guaranteed to fall inside a safe operating zone, with similar optimization and prediction results. Baseline separation of the enantiomers was obtained quickly during 3 min, with a resolution higher than 1.5 [75]. The study confirms the principle that the applied three response surface designs can serve as alternatives to each other.

A CE chiral separation method was developed by Danel et al. for the enantioseparation of 9-hydroxyrisperidone, the main active metabolite of risperidone, an antipsychotic drug. A dual CD system was employed, composed of neutral HP-β-CD and anionic S-α-CD at an acidic pH of 2.5. A CCD was used for method optimization, selecting experimental factors as: HP-β-CD concentration, S-α-CD concentration and BGE concentration; the analytical responses were chiral resolution, migration time, selectivity, and plates number. The influence of temperature and voltage was studied using a OFAT strategy, however, both parameters had a great impact on the migration time and the chiral resolution as well. Probably the inclusion of these two factors in the multivariate optimization could be useful for detecting possible interactions with the other factors. The method was applied for the simultaneous determination of risperidone and 9-hydroxyrisperidone enantiomers, and it could be used for the determination of analytes in biological samples, to characterize risperidone metabolism [76].

An experimental design strategy was used by Sungthong et al. for the development of a CE stereoselective method for the simultaneous determination of *S*-citalopram chiral impurities, including its distomer, *R*-citalopram, and a synthetic intermediate, *S*-citadiol. Citalopram is a selective serotonin reuptake inhibitor (SSRI) antidepressant, used in therapy first in the form of racemate and then as pure enantiomer, *S*-citalopram, being a successful example of “chiral switch” among pharmaceuticals. After an initial CD screening, a dual CD system composed of native β-CD and anionic S-β-CD was chosen as the optimum CS for the separation. A FCCD was used in the study; the influence of four factors, BGE concentration, S-β-CD concentration, voltage, temperature over the chiral resolutions between the four enantiomers, migration time of the last migrating enantiomer and generated current was evaluated. BGE pH was set at 2.5 as the analytes are basic, and the concentration of the second CD, β-CD, was kept constant during optimization, based on preliminary results. The method was validated and allowed the detection of the impurities at a 0.05% level relative to *S*-citalopram at a concentration of 5 mg/mL. The assay was used to check the purity of *S*-citalopram in both bulk samples and tablets [77].

A BBD was used by Borges et al. for the development of a CE chiral separation method for the simultaneous enantioresolution of propranolol and its main active metabolite, 4-hydroxypropranolol. CM-β-CD was used as CS in a triethylamine/phosphoric acid BGE at a basic pH. The influence of four factors, namely BGE concentration, BGE pH, CM-β-CD concentration, and voltage on chiral resolution and migration time, was studied. The method has the potential to be used in quality control of pharmaceutical formulations, but also in pharmacokinetic and metabolic studies [78].

Gomis et al. developed a CE chiral separation method for the enantioseparation of zopiclone, a sedative-hypnotic substance and one of its synthesis precursors. For optimization, a FFD was used; four factors were selected, BGE concentration, BGE pH, β-CD concentration and voltage, and their influence on chiral resolution and migration time was studied. The results of the method, compared with a previously published HPLC method, proved CE to be superior in terms of number of theoretical plates, migration time and cost-effectiveness. The method can be used to evaluate the presence of chiral synthesis intermediates and the enantiomeric purity of zopiclone [79].

A CE method for the separation and determination of clopidogrel impurities was developed by Fayed et al. Clopidogrel is an anti-platelet dihydrothienopyridine derivative prodrug, used in therapy in the form of a pure enantiomer, *S*-clopidogrel; in this study the presence of the distomer, *R*-clopidogrel and two other chemically related substances was investigated. A reduced CCD was used to study the effects of BGE concentration, BGE pH, S-β-CD concentration and voltage on the chiral resolution and migration time. The method was validated and used for *S*-clopidogrel purity control in bulk commercial samples [80].

Liu et al. developed a CE method for enantiomeric purity control of RS86017, a potential antiarrhythmic drug with two chiral centers using SBE-β-CD as CS. BGE concentration and pH, SBE-β-CD concentration, organic modifier concentration, temperature, and voltage were systematically optimized by OAD and CD concentration was further optimized using a OFAT strategy. The optimized method was validated and was capable of separating the *R*,*S*-enantiomer of RS86017 from its potential chiral impurities, *S*,*R*-enantiomer, *R*,*R*-diastereomer and *S*,*S*-diastereomer, in a relatively short analysis time of 10 min. The values of limit of detection (LOD) and LOQ were 0.8 µg/mL and 2.5 µg/mL, respectively for all enantiomers. The procedure can be useful for the quality control of the pure enantiomeric drug product [81].

Chiral separation by CE of dapoxetine, a serotonin transporter inhibitor for the treatment of premature ejaculation has been achieved by Neumajer et al. using randomly methylated γ-CD (M-γ-CD) as CS. Dapoxetine is used in therapy in the form of pure enantiomer, *S*-dapoxetine. To establish the optimum CS a wide range of CDs has been tested at various concentration levels, also the apparent binding constants were calculated. An OAD was used to optimize experimental parameters; the influence of BGE concentration, BGE pH, M-γ-CD concentration, organic additive (methanol) concentration, temperature, voltage on the chiral resolution was investigated. The method was validated, and a PBD was applied for robustness testing [82].

A few years later, Harnisch and Scriba published a CE method for the determination of *S*-dapoxetine chiral purity regarding its distomer, *R*-dapoxetine and another chemical-related impurity. An initial CD screening at two concentration levels and three pH levels was applied in the preliminary analysis. A dual CD system containing DM-β-CD and sulfated-γ-CD (S-γ-CD) was used in the separation. For the optimization of the analytical conditions, a FrFD was used for screening followed by a CCD with Monte Carlo simulation to define the design space. The robustness of the method was verified by a PBD. The method was validated and applied for analysis of dapoxetine in tablets. The method was able to determine the impurities at a level as low as 0.05%. The results of the CE assay for enantiomeric purity of dapoxetine were comparable to those of an enantioselective HPLC approach [83]. The assay is an example of how CE may be used to evaluate related compounds and enantiomeric impurities in a single run.

Zhang and Du applied two new amino acid ionic liquids as CS in the enantioseparation of pharmaceuticals and evaluated their potential synergistic effect with glycogen. Three chiral model molecules were used in the study (citalopram, duloxetine, nefopam). When compared to the results achieved involving only glycogen as the CS, the chiral ionic liquids/glycogen synergistic systems significantly enhanced enantiomer separation. Several major enantioseparation factors, such as the concentration of amino acid ionic liquids, glycogen concentration, and BGE pH, were extensively studied in a univariate way; however, considering the aim of this study a multivariate approach could be particularly useful to detect interactions between ionic liquids and glycogen. The influences of three other parameters, BGE concentration, voltage, and temperature, were simultaneously assessed by a CCD to further improve the overall synergistic system [84].

Asensi-Bernardi et al. proposed a modelling strategy to assess the chiral separation ability of highly sulphated-β-CD (HS-β-CD) as CS. In this study a discriminant partial least squares (PLS)-based quantitative structure-property relationship (QSPR) method was simplified, being translated into an explicit equation that may predict the enantioresolution of new analytes using four structural parameters found in open databases: the logarithm of the octanol-water partition coefficient calculated at pH 7.4, polar surface area, number of hydrogen bond donors, and acceptors. In the situations when the model predicted enantioresolution, a BBD was proposed for fast PLS-based optimization using as experimental design factors: BGE pH, CD concentration and temperature [85]. This study is a good example of how different theoretical and computational approaches can work in a complementary way to reduce the number and the cost of experiments during method development.

A chiral CE method using a dual CD system composed of a cationic and neutral CDs was developed for the enantiomeric separation of a compound presenting a diaryl sulfonamide group by Rogez-Florent et al. A dynamic cationic coating of the capillary was used to prevent adsorption of the amino-β-CD to the capillary wall. The method was optimized using a CCC design with three experimental factors: amino-β-CD and β-CD concentrations and methanol percentage in the BGE. The design results showed a significant interaction effect of the two CDs on chiral resolution [86].

Wahl and Holzgrabe developed a CE method for the evaluation of enantiomeric purity of magnesium-*L*-aspartate dihydrate. A CE method using HP-β-CD coupled to laser induced fluorescence (LIF) detection and a HPLC-fluorescence method using chiral derivatization by o-phthaldialdehyde and N-acetyl-L-cysteine were developed and validated. A three level FFD was used to investigate the influence of the effects of BGE concentration, BGE pH and CD concentration, on the resolution between *L*- and *D*-aspartic acid. The method was used for the determination of *D*-aspartic acid content in bulk samples and dietary supplements [87].

QbD concepts were applied for the analysis of levosulpiride, an atypical antipsychotic in a study by Orlandini et al. A dual CD system composed of S-β-CD and M-β-CD was used as CS in the separation. By conducting a screening design comprising both qualitative (neutral CD type) and quantitative (BGE concentration, BGE pH, neutral and ionized CD concentrations, voltage) factors, as well as a response surface investigation, this separation system was thoroughly explored using multivariate approaches. The qualitative factor was studied at two levels, while the quantitative factors were all assessed at three levels. RSM was carried out using a DD to highlight significant interactions between critical process parameters. Design space was defined by applying Monte Carlo simulations. A PBD was applied for robustness testing. The method was validated and applied for the determination of enantiomeric purity of levosulpiride in pharmaceutical dosage forms [88]. To the best of our knowledge, this was the first time a QbD approach has been used to systematically determine enantiomeric purity in a pure enantiomer medication. The use of a QbD approach in the development of a CE methods allowed for a more reasonable and systematic approach to optimization difficulties, providing for a better understanding of the chiral separation.

The enantiomers of asenapine, a novel antipsychotic agent with a dibenzooxepino pyrrole structure used in schizophrenia, were separated by CE by Szabó et al. Asenapine has two chiral centers in its structure, however, it is used in therapy in the form of a racemate composed of *R*,*R*- and *S*,*S*-enantiomers. The stability of the inclusion complexes and the enantiodiscriminating ability were investigated with different CDs, showing β-CD as the most promising. An OAD was used for method optimization, varying in a multivariate manner the BGE concentration, BGE pH, β-CD concentration, organic modifier (methanol) concentration in the BGE, temperature and voltage; the response being the chiral resolution. Molecular modelling, NMR spectroscopy, and ESI–MS were used to identify the complex’s stoichiometry and shape. The analyte-CD complex was found to have 1:1 stoichiometry, and either of the two aromatic rings may be fitted in the CD cavity [89].

The same research group published a CE chiral separation method for the enantioseparation of pomalidomide, a thalidomide derivative used as an immunomodulatory drug. As the drug is neutral, ionizable CDs were used in the screening to establish the optimum CS; CM-β-CD was found to be the most efficient. An OAD was used to optimize BGE concentration, BGE pH, temperature, and voltage; chiral resolution was selected as analytical response. The method was validated and allowed the baseline separation of the enantiomers with high resolution values [90].

In a study by Szabó et al., different CDs and polysaccharides were used as CSs, in two complementary methods (CE, HPLC) to investigate enantiomeric resolution of lenalidomide, an immunomodulatory agent. HPLC separation was achieved using a polysaccharide type chiral stationary phase (CSP) in polar organic (PO) mode. In the case of CE separation, after a preliminary screening of several anionic chargeable CDs, SBE-β-CD was chosen as optimum CS. An initial OFAT strategy was applied for method optimization, followed by a FCCD for fine-tuning the separation. The method was validated and allowed the determination of 0.1% *R*-lenalidomide [91].

Chiral separation of vildagliptin by CE, an oral antidiabetic from the DPP-4 inhibitor class, was reported by Kazsoki et al. Vildagliptin is used in therapy in the form of pure enantiomer, *S*-vildagliptin, *R*-vildagliptin being considered as its chiral impurity. After a complex screening of 13 CDs, SBE-β-CD was chosen as optimum CS. Optimization was performed using an OAD; the influence of BGE concentration, BGE pH, SBE-α-CD concentration, temperature, voltage, and injection parameters on chiral resolution was verified. After the multivariate optimization, it was observed that the second peak (*S*-vildagliptin) migrated too close to the electroosmotic flow (EOF), which would influence chiral purity control. To fine-tune the optimization, in a second step the OFAT approach was applied. However, as an assumption, if the Authors had used first the OFAT method to identify the conditions in which the vildagliptin enantiomers were separated and migrated far from EOF, and after that the OAD, this flaw may have been avoided. Another possibility could also be to include the time distance of the peak, from EOF, as the second response to be directly evaluated by OAD. The method was validated; LOD and LOQ values were 2.5 and 7.5 µg/mL, for *R*- and *S*-vildagliptin, respectively. The robustness of the method was verified using a PBD. The method was applied to control the chiral purity of *S*-vildagliptin in pharmaceutical formulations [92].

A CE method for the determination of chiral purity of ambrisentan was published by Krait et al. Ambrisentan is an orphan drug used in the form of pure enantiomer, *S*-ambrisentan, as a selective endothelin receptor antagonist for the treatment of pulmonary arterial hypertension. γ-CD was used as CS in the enantioseparation. The approach was developed using a FrFD to identify the significant factors followed by a FCCD response surface approach and Monte Carlo simulations to generate the DS. PBD was used for robustness testing. The method was validated and allowed the determination of 0.1% concentration of enantiomeric impurity *R*-ambrisentan [93].

A CE method for the determination of three chiral impurities of sitafloxacin, a chemotherapeutic fluoroquinolone, was developed by Meng and Kang. Sitafloxacin has three chiral centers, which generates the presence of eight stereoisomers; however, due to stereoselective synthesis at least two pairs of enantiomers (four stereoisomers) may be present in the bulk substance. A dual CS system containing γ-CD and Cu^2+^-*D*-phenylalanine was used; the combination of the two CSs enhanced stereoselectivity through the combination of separation mechanisms: inclusion-complexation (CD) and ligand exchange (Cu^2+^-*D*-Phe). A FrFD was used in the screening, involving seven factors, and a FCCD was used in the optimization, studying the influence of BGE pH, γ-CD, Cu^2+^, *D*-Phe concentrations on chiral resolution and migration time. Concentration of impurities as low as 0.1% with respect to sitafloxacin eutomer can be detected using the validated method [94].

CE chiral separation of fluoxetine, a SSRI antidepressant, was reported by Cârcu-Dobrin et al. Fluoxetine is used in therapy as a racemate, however, its metabolism is stereoselective, making chiral methods useful in pharmacokinetics studies. To establish the optimum CS, a vast variety of native and derivatized, neutral and ionized CD derivatives were examined at three pH levels; heptakis (2,3,6-tri-O-methyl)-β-CD (HTM-β-CD) at pH 5.0 was chosen for enantiomeric differentiation. An OAD was applied in the optimization process: the influence of BGE concentration, BGE pH, CD concentration, temperature, voltage, and injection parameters on enantioresolution, was tested. The method was validated and applied for the enantioseparation of fluoxetine in pharmaceuticals [95].

A CE method for the determination of chiral purity of pregabalin upon derivatization with dansyl chloride was developed by Harnisch et al. Pregabalin, a γ-aminobutyric acid analogue, is used in the form of pure enantiomer, *S*-pregabalin, as anticonvulsant and anxiolytic. HTM-β-CD was used as CS. Initially a FrFD design was applied, however peak tailing was detected at low BGE concentrations; when BGE concentration was increased, the peak tailing impact was minimized. As a result, the experimental zone had to be changed to incorporate larger BGE concentrations, resulting in an asymmetrical experimental zone. Thus, a D-oD was used for screening to identify significant parameters, while a FCCD and Monte Carlo simulations were employed for further optimization and to define the DS of the method, respectively. The robustness of the method was tested using a PBD. The method was validated and allowed the detection of *R*-pregabalin at the 0.015% level with a LOQ at the 0.05% level in a sample containing 1.59 mg/mL pregabalin [96].

A CE method has been developed according to QbD principles by Krait and Scriba for the chiral purity determination of dexmedetomidine. Dexmedetomidine is a selective α_2_-adrenergic agonist used as general anesthetic in the form of a pure enantiomer. The analytical target profile was set to determine the distomer levomedetomidine, with appropriate precision and accuracy at 0.1% level. After an initial CD screening, S-β-CD was selected as CS. A FrFD was utilized to identify the significant process parameters, followed by a FCCD as optimization design and Monte Carlo simulation to define the method’s design space. The robustness of the method was assessed using a PBD. The method was validated and applied for the determination of dexmedetomidine in tablets [97]. For the identification of the critical process parameters (CPPs), which are the experimental factors that impact the method’s critical quality attributes (CQAs), this methodology uses risk-assessment tools. This approach leads to a quantitative knowledge of the probability for the requirements for CQAs to be fulfilled.

**Figure 1:**
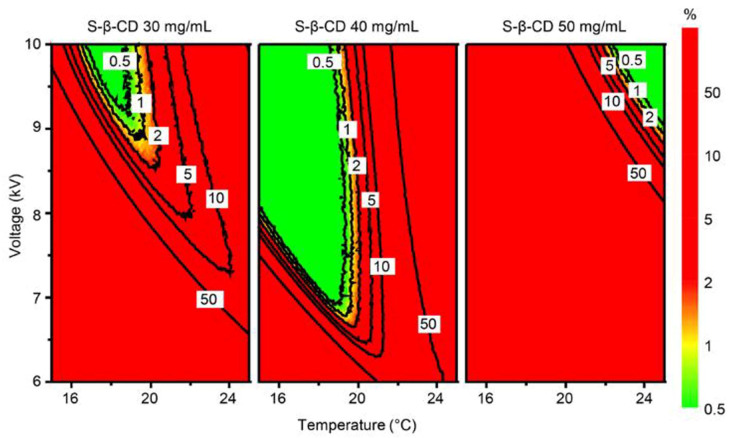
Probability plot of the design space obtained by Monte Carlo simulations in the case of dexmedetomidine. Reprinted from Krait and Scriba [97] with permission from Wiley.

From the article mentioned above we selected Figure 1 which shows the plots obtained with Monte Carlo simulation for concentrations of S-β-CD of 30, 40 and 50 mg/mL; the design space (green zone) defines combinations of parameters where the risk of failure is below 1%, to meet the three selected criteria, separation factor-value above 1.05, generated current below 100 µA, and migration time of dexmedetomidine below 10 min. It can be observed that the larger space is found in the case of 40 mg/mL S-β-CD concentration [97].

The same researchers developed a chiral CE implementing QbD principles for the determination of dextromethorphan chiral purity using a dual selector system, composed of methyl-α-CD (M-α-CD) and S-β-CD. Dextromethorphan is a centrally acting antitussive medication, but its enantiomer, levomethorphan, is an illicit opioid analgesic. A FrFD was used to assess the significant analytical parameters, followed by a FCCD and Monte Carlo simulations to define the method’s DS. After selecting the optimum working conditions, a PBD was used to assess the method’s robustness before it was validated. To understand the migration behavior of the analytes, the apparent binding constants between the enantiomers and the CDs, as well as complex mobilities, were calculated. The method was used to determine the stereochemical impurity levomethorphan in dextromethorphan bulk substance and pharmaceutical formulations at 0.1% level [98].

Pasquini et al. applied QbD principles for the determination of chiral purity of *R*-cinacalcet, a calcimimetic drug, and two other related chiral impurities. A preliminary CD screening was applied to establish the optimum CS, and HP-γ-CD was selected as the best solution. A BBD was applied for method optimization; BGE pH, HP-γ-CD concentration, methanol concentration and voltage were selected as variables; their effect on chiral resolution and migration time was investigated. The mathematical model developed by using BBD allowed for a more in-depth knowledge of the response behavior; the researchers focused on the conditions that would allow to achieve higher resolution of enantiomers and the synthesis impurities and lower migration times. Using Monte-Carlo simulations, the MODR was found as the multidimensional zone where both critical method attributes satisfied the requirements with a desirable probability. The QbD methodology aided in determining the experimental zone in which the likelihood of failing to meet the previously stated criteria was lower than 10%. A PBD was used to verify robustness; the method was validated and applied for cinacalcet determination in pharmaceuticals [99].

Vargas-Martínez and Ramírez-Galicia developed a CE method for the enantioseparation of four chiral benzodiazepines (lorazepam, lormetazepam, oxazepam, temazepam). Three CDs were tested: heptakis-6-sulfato-β-CD (HES-β-CD), heptakis(2,3-di-O-acetyl-6-O-sulfo)-β-cyclodextrin (HDAS-β-CD), heptakis(2,3-di-O-methyl-6-O-sulfo)-β-cyclodextrin (HDMS-β-CD); the best results were obtained when using HES-β-CD. A 3^4-2^ FrFD was applied in the optimization; CD type, CD concentration, BGE pH, percentage of methanol in the BGE were selected as factors. The apparent equilibrium constants for the formation of the benzodiazepine-CD complexes were calculated, and a theoretical investigation of the interaction between the benzodiazepine and the HES-β-CD complex was proposed using semiempirical calculations [100].

Wahl and Holzgrabe proposed the application of ionic liquids combining tetrabutylammonium cations with chiral amino acid-based anions as BGE additives for the enantioseparation of phenethylamine (ephedrine, methylephedrine, pseudoephedrine) enantiomers, while β-CD was used as a CS. BGE pH, and ionic liquids concentration were optimized using a three level FFD [101].

An interesting study using DoE, in which one of the major drawbacks of CE, transfer of CE methods, is discussed was published by De Cock et al. A further weakness of CE is the lower sensitivity when compared to HPLC; improving and maintaining sensitivity is the reason why inter-instrumental differences across detector settings should be prioritized. The chiral separation of two β-blockers (betaxolol, propranolol) was considered as a case study. The influence of several detector parameters, including data acquisition rate, bandwidth, and filter, on several responses (peak area, height, and width, signal-to-noise ratio, peak asymmetry, migration time, efficiency, resolution), was evaluated by robustness testing performed on two CE instruments [102]. This research is part of a wider initiative that aims to provide standards for transferring CE procedures between different devices or laboratories.

A generic CE method was proposed by Abdel-Megied et al. for the chiral enantioseparation of different basic and acidic drugs; anti-Alzheimer (donepezil, rivastigmine) and antimycotic (fluconazole, itraconazole, ketoconazole, sertaconazole) drugs were considered as model solutes. Several neutral and charged CDs were tested as CSs. An initial OFAT study was approached for screening purposes followed by a FFD using two significant factors (BGE pH and CD concentration) at three levels for optimization. The best results were obtained when using SBE-β-CD at low pH values and high CD concentration [103]. However, the regression results indicated that the linear model shows a significant lack of fit for all CDs, implying that higher orders of the factors are most likely to be included in the equation with possible interactions.

Two chiral separation CE methods for the enantioseparation of proton pump inhibitors (lansoprazole and rabeprazole, respectively) were developed by Papp et al. A high number of neutral and ionized CDs were screened at two pH levels to identify the optimum CS, and SBE-β-CD was selected as the best option. Furthermore, various dual CD systems were tested, and possible separation mechanisms were studied; a dual CD system containing SBE-β-CD and γ-CD was finally considered to be the most adequate for the enantioseparation. An initial FrFD was used for identifying significant experimental factors followed by CCD to establish optimum analytical conditions. The method was validated and used for the determination of 0.15% distomer in dexlansoprazole and dexrabeprazole samples [104].

CE chiral separation of tramadol, a centrally acting analgesic drug, was reported by Sarkany et al. Tramadol has two chiral centers which generates four stereoisomers, however, it is used in therapy as a racemic mixture of the trans enantiomers, *R,R*- and *S,S*-tramadol. Charged and uncharged CDs were screened at four pH levels to establish the optimum CS; CM-β-CD in a basic BGE was selected as the best solution. After a preliminary OFAT approach a FCCD was used to establish the optimum electrophoretic conditions. The analytical performance of the method was verified, and the method was applied for the determination of enantiomer ratio of tramadol in pharmaceutical preparations [105].

**Figure 2:**
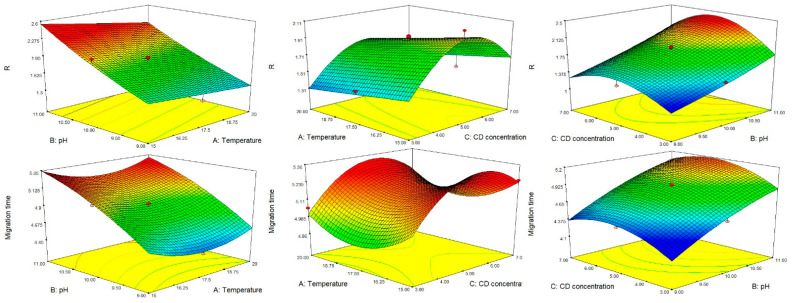
3-D response surface plots for the chiral separation of tramadol. Reprinted from Sarkany [105] with permission from Springer.

From the article mentioned above we selected Figure 2 which presents 3-D response surface plots representing the influence of three significant factors (pH, CD concentration, temperature) on the two selected analytical responses (chiral resolution, migration time).

Niedermeier and Scriba published also a QbD guided method development for the determination of dextrodropropizine and 1-phenylpiperazine as impurities in levodropropizine samples. Levodropropizine is a non-opioid peripherally acting drug used as antitussive, in the form of pure enantiomer. Initially, a CD screening at three pH levels testing a large number of neutral and anionic CD derivatives was performed and eventually S-β-CD was selected as optimum CS. A FFD was used to study the effect of CD concentration, temperature, voltage, and 2-propanol concentration on the selected CQAs of the method (separation factors, migration time, generated current). The following method optimization consisted of a two-factor FCCD for further studying CD concentration and temperature, then the design space was determined by Monte Carlo simulations. An additional robustness testing was carried out using a PBD before the method was validated and applied for the analysis of levodropropizine bulk samples and pharmaceutical drops [106]. This study is a good example of the simultaneous determination of chiral and achiral impurities of an enantiopure drug.

Another CE chiral separation method for the enantioseparation of amlodipine was developed by Cârcu-Dobrin et al. CM-β-CD in a basic BGE was selected as CS, based on an initial CD screening at four pH levels. An OAD was used for method optimization, examining six experimental factors: BGE concentration and pH, CM-β-CD concentration, temperature, voltage, and injection parameters. The analytical performance of the method was verified, and the method was applied for the determination of amlodipine in pharmaceutical preparations [107].

The enantioseparation of citalopram was published by Budău et al., based on an initial CD screening at four pH levels; CM-β-CD was selected as optimum CS. For method optimization, an experimental design approach was utilized: a FrFD was applied for screening to identify significant experimental parameters, followed by a FCCD for optimization. To help in the understanding of the chiral separation mechanism, computational modelling was employed allowing to gain information on the interaction energy and shape of the analyte-CD complexes. The method was applied for the determination of enantiomers in racemic formulations and optical purity control of *S*-citalopram [108].

The same research group worked on a CE method for the enantioseparation of venlafaxine published by Milan et al. Venlafaxine is an antidepressant from serotonin and norepinephrine reuptake inhibitor (SNRI) class, used in therapy as a racemate, however differences between pharmacological profiles of the enantiomers are known. CD screening at four pH levels led to the conclusion that CM-β-CD in an acidic BGE is the optimum CS. An initial OFAT screening technique was utilized to determine the impact of analytical parameters on the enantioseparation, followed by an optimization procedure using a FCCD. To describe host-guest chiral recognition, computer modelling of venlafaxine-CD complexes was applied. The method was validated and used for the determination of venlafaxine enantiomers in pharmaceutical formulations [109].

### 3.2. Capillary Electrophoresis—Mass Spectrometry (CE-MS)

One of the few chiral CE-MS methods developed by DoE was published by Rudaz et al. for the enantioseparation of methadone, a synthetic opioid agonist. A capillary electrophoresis coupled with electrospray ionization mass spectrometry (CE-ESI-MS) was used. A partial filling technique was used to avoid the CS entering the MS ion source. Three different CDs: HP-β-CD, CM-β-CD and SBE-β-CD were tested as CSs; a volatile BGE composed of ammonium acetate at pH 4.0 was used. Initially, an FFD was used to investigate the significance of selected factors, and some center point measurements were also performed to assess the curvature of the model. Since significant non-linear terms were found, the FFD was completed with axial points to obtain a FCCD. The same type of experimental matrix was performed in the case of all the three CDs. CD concentration, percentage of capillary filled with CS and drying gas nebulization pressure were the factors taken into consideration; chiral resolution, apparent selectivity and migration time were set as responses. CD concentration and gas nebulization pressure had a significant effect on the quality of the separation. Response surfaces were drawn from the mathematical model and optimum experimental conditions were set to allow the robustness of the method [110].

### 3.3. Micellar Electrokinetic Chromatography (MEKC)

In MEKC the separations are achieved in the presence of surfactants supplemented to the electrolyte solution at micellar concentration. Using ionic surfactants, the applicability of CE is extended to neutral molecules thanks to their differential distribution between the ionic micellar phase and the aqueous phase. When achiral surfactants are used, that is most of the cases, direct enantioseparation can only be achieved including the CS to the micellar BGE. The obtained mixed organized media represent intriguing systems because of the possibility for multiple interactions i.e., between the solute enantiomers with the CS and the micellar phase as well as between the latter and the CS. In these complex systems, including in DoE variables such as nature and concentration of the surfactant, is of primary importance.

Wan et al. applied two approaches for the chiral separation of 19 amino acids by CE; an indirect separation of diastereomers formed by derivatization with (+)- or (−)-l-(9-fluorenyl)ethyl chloroformate (FLEC—used as derivatization agent) and a direct chiral separation after derivatization with 9-fluorenylmethyl chloroformate (FMOC). In both cases, MEKC methods were applied for the separation. The analytical conditions were optimized using an FFD. In the indirect separation, sodium dodecyl sulphate (SDS) concentration and BGE pH, while in the direct separation isopropanol concentration, β-CD concentration and SDS concentration were optimized using the experimental design. The analytical performances of the indirect method proved to be superior, offering higher separation efficiency [111].

Orlandini et al. developed a CE chiral separation method for the simultaneous determination of the enantiomeric purity of ambrisentan, including three additional achiral impurities. A MEKC method was applied, using SDS as surfactant and γ-CD as CS. The effect of BGE concentration, BGE pH, γ-CD concentration, SDS concentration, temperature, voltage and capillary length on chiral resolution and analysis time was investigated using a screening asymmetric matrix. A FCCD was further used for optimization purposes, studying the effect of significant factors (BGE pH, γ-CD concentration, voltage) on the separation. The CE approach was developed inside the QbD framework, with the goal of creating a DS where analysis results fulfil predetermined quality features with a certain degree of probability. The DS was defined in combination with Monte-Carlo simulations. The robustness of the method was verified using a PBD. The method was validated and applied for the determination of ambrisentan in coated tablets [112]. The method can be highlighted because it is an example of how CE can be used for the simultaneous determination of enantiomeric purity and of related substances of a pure enantiomer drug.

A MEKC method was developed by Flor et al. for the chiral purity control of montelukast, a leukotriene antagonist receptor used in the treatment of asthma. The method allows the simultaneous determination of *R*-*trans*-montelukast, its distomer (*S*-*trans*-montelukast), diasteroisomers (*R*,*S*-*cis* forms), and its main degradation compound, montelukast sulfoxide. A dual CD system composed of HTM-β-CD and SBE-β-CD was employed in the separation. In the optimization step, the influence of BGE pH and voltage was carried out using a FFD with two factors at three levels, followed by analyzing the temperature by a OFAT approach. This was an uncommon optimization strategy since it was carried out in two steps: in the first, the optimization was performed using a multivariate technique, and in the second, using a univariate technique. Moreover, if temperature had been taken into consideration in the original FFD, the algorithm may have used a multivariate technique to execute the optimization stage. Moreover, important factors for enantioseparation as CS concentration have not been selected for the multivariate approach. Robustness was evaluated using a PBD. The method allows the determination of 0.02% enantiomeric and diasteroisomeric impurities, and 0.01% montelukast sulfoxide. The method was used for the determination of chiral purity of montelukast in bulk substances and pediatric pharmaceutical forms [113].

Niedermeier and Scriba developed a CE method for the simultaneous determination of the chiral impurity dextromepromazine and the oxidation product levomepromazine sulfoxide in levomepromazine samples. Levomepromazine possesses an *R*-configuration and is used as pure enantiomer as antipsychotic. Hydroxypropyl-γ-CD (HP-γ-CD) was the selected CS and a FrFD was used for the optimization of BGE concentration, BGE pH, HP-γ-CD concentration, temperature, and voltage. A FCCD was applied as response surface methodology for deriving the design space by Monte Carlo simulations. Robustness was estimated by means of a PBD. The method was applied for the determination of the analyte in a commercial injection solution [114].

### 3.4. Non-Aqueous Capillary Electrophoresis (NACE)

In NACE pure organic solvents or their mixtures are employed in the preparation of running BGE, to improve the solubility of hydrophobic analytes and/or to acquire particular selectivity due to the effect of the solvents on the solutes’ acid-base characteristics. There are some specific constraints in the development of chiral NACE methods, e.g., the CS has to be soluble in the selected solvent and its ability to establish interactions with the solutes, must not be compromised by the presence of the organic solvent. This could be the case of CDs whose hydrophobic cavity can be overridden by the organic solvent and no longer accessible to the solutes. On the other hand, in NACE, often solvents with lower dielectric constants with respect to water, are used thus favoring the interactions between the entities involved in the separation process. Under these conditions, the (enantio)selectivity can be favorably tuned by the use of additives as supplementary components of the BGE.

Servais et al. studied the influence of the nature of the cationic (ammonium, potassium, sodium) and anionic (chloride, formate, camphorsulfonate, methanesulfonate) BGE components as well as the concentration of HDMS-β-CD on the chiral resolution of 6 basic analytes (three β-blockers: atenolol, celiprolol, propranolol; three local anesthetics: bupivacaine, mepivacaine, prilocaine) using NACE. Two D-oDs were used in the optimization process. Both cationic and anionic BGE components had a significant impact on the enantiomeric resolution of the analytes, although the cationic component exerted the greatest influence. A NACE system was recommended for the separation, namely ammonium formate and potassium camphorsulfonate in a methanolic solution containing HDMS-β-CD and acidified with formic acid [115].

NACE was applied by Marini et al. for the determination of *R*-timolol as chiral impurity in *S*-timolol maleate samples. Timolol is the only β-blocker used in therapy as pure enantiomer, in the form of *S*-timolol, in the treatment of glaucoma. DoE was used for robustness testing and uncertainty assessment from quantitative data. The study was based on the results of a previously published method by Marini et al., in which timolol enantioresolution was achieved using HDMS-β-CD as CS and potassium camphorsulphonate in methanol acidified with formic acid as BGE [116]. One qualitative and six quantitative parameters were investigated using a PBD. Resolution, migration times, and relative migration times to pyridoxine (internal standard) were investigated as qualitative responses for electrophoretic performance, while the content of *R*-timolol in *S*-timolol maleate sample was evaluated as a quantitative response. The selected parameters had no significant effect on the quantitative result, indicating the procedure’s robustness; however, diverse HDMS-β-CD batches have been observed to have an impact on both qualitative and quantitative outcomes [117].

In another study, the separation of 10 β-blockers (acebutolol, atenolol, betaxolol, celiprolol, metoprolol, oxprenolol, pindolol, propranolol, sotalol, timolol) using HDMS-β-CD and HDAS-β-CD as CS was investigated by Rousseau et al. A D-oD was applied in method development; the experimental design involved two quantitative (BGE and CD concentration) and two qualitative (BGE anion and CD nature) factors; while BGE counter-ion, temperature, voltage, and formic acid concentration were kept constant. Chiral resolution, mobility difference and selectivity were selected as analytical responses. Both CD type and concentration were shown to have a substantial impact on the enantiomeric resolution for all analytes. HDAS-β-CD was found to have a higher enantioresolution ability than HDMS-β-CD for most analytes. All compounds had the best enantioseparation in the presence of a high CD concentration, and most of them in the presence of a low BGE anion concentration. A generic NACE system using methanol as the solvent, was proposed for the enantioseparation of all analytes; to confirm the suitability of the technique, the optimal experimental conditions were compared to the conditions obtained by modelling mobility difference and selectivity [118].

The same research group published later a study regarding the development of a generic CD system for the enantioseparation of basic drugs by NACE in acidifies methanol, using heptakis(2-O-methyl-3-O-acetyl-6-O-sulfo)-β-CD (HMAS-β-CD) as CS. Four imidazole antifungal agents (econazole, isoconazole, miconazole, sulconazole), three local anesthetics (bupivacaine, mepivacaine, prilocaine), two sympathomimetics (salbutamol and terbutaline) and one β-blocker (carvedilol) were selected as basic model analytes. The effect of CD and BGE anion concentrations, as well as the type of the BGE anion, on the chiral resolution was examined by D-oD; CD concentration was shown to have a substantial impact on the chiral resolution. A NACE system based on HMAS-β-CD was proposed and compared to both previous systems using HDAS-β-CD or HDMS-β-CD. A fast method development strategy based on HMAS-β-CD was applied for the enantioseparation of ketamine, and its metabolite norketamine, after in vitro metabolism (incubation of ketamine in phenobarbital-induced male rat liver microsomes systems) [119].

Niedermaier and Scriba developed a NACE method using methanol as the medium for the chiral separation of four phenothiazine derivatives (alimerazine, mepromazine, promethazine, thioridazine). HDAS-β-CD, HDMS-β-CD and octakis(2,3-di-O-methyl-6-O-sulfo)-γ-CD (ODMS-γ-CD) proved to be effective CSs for mepromazine, promethazine and alimemazine, while thioridazine was only partially resolved by using SBE-β-CD. In the same study a NACE method for the determination of dextromepromazine as chiral impurity of levomepromazine was developed employing QbD principles. HDMS-β-CD was used as CS and a FrFD was developed for evaluating the knowledge space, followed by a FCCD for further method optimization; the DS was configured using Monte Carlo simulations. Robustness of the method was verified using a PBD. The validated method was applied in chiral purity determination of levomepromazine in bulk substance and tablets. The assay was used also to detect levomepromazine sulfoxide, albeit quantification was impeded by the second migrating diastereomer’s poor peak shape [120].

### 3.5. Capillary Electrochromatography (CEC)

In CEC the capillary is filled with a stationary phase; once the selected electrolyte solution is loaded and an appropriate conditioning is performed, the applied electric field results in EOF which will pump the electrolyte solution through the capillary column similarly to the mobile phase in HPLC. The analytes are separated according to the combined effects of the interactions with the chromatographic phase and on the basis of their electrophoretic mobility.

Chiral separation of 25 model compounds (neutral, acidic, basic) was carried out by Karlsson et al. using aqueous and non-aqueous CEC and a glycopeptide antibiotic, teicoplanin, as CSP. The impact of non-aqueous PO mobile phase parameters on the EOF, chiral resolution, and peak efficiency was evaluated using a DoE strategy for two model compounds (metoprolol, terbutaline); the methanol content of the mobile phase proved to be the most important factor for obtaining high values of the selected responses. Teicoplanin CSP proved to be a good solution in CEC in reversed phase (RP) and PO conditions for the enantioseparation of a high number of pharmaceuticals [121].

Wikström et al. published a CEC study, in which another glycopeptide antibiotic, vancomycin, was used as CSP covalently bonded to LiChrospher® diol silica packed column both in the RP and PO mode. A DoE strategy was used to determine the optimum PO phase composition, which indicated that organic modifier content ratio (methanol, acetonitrile) has a significant effect on resolution and peak efficiency. Thalidomide was considered as model molecule during the study; after optimization, the method was used to separate several basic analytes including four β-blocking agents (alprenolol, atenolol, metoprolol, practolol) in PO mode [122].

A generic experimental strategy for the enantioseparation of 15 acidic drugs by CEC using polysaccharide CSPs was developed by Mangelings et al. Four CSPs were tested: Chiralcel OD-RH, Chiralcel OJ-RH, Chiralpak AD-RH and Chiralpak AS-RH. The studied factors included BGE concentration, BGE pH, acetonitrile content of the mobile phase, voltage, temperature; chiral resolution and analysis time were set as responses [123]. Starting with a general screening experiment, the study was built on a concept of a decision tree. A screening process for the four CSPs was established, based on the idea that majority of enantioselectivity will be visible in the initial trial. A three-level four-factor well-balanced design derived from a PBD and proposed by Vander Heyden et al. was used [124]. However, the addition of voltage to the initial design as the fifth factor increased the number of experiments from 9 to 27. A proper choice of the screening design (FrFD) could probably have provided the same efficiency but in fewer experiments. The authors pointed out the fact that if baseline separation is seen during screening, an optimization phase could be useful to enhance resolution and/or reduce analysis time.

Table 1 presents chronologically a list of chiral CE methods for the analysis of certain pharmaceuticals developed by using DoE strategies.

## 4. Discussion

Given the advantages of using DoE strategies in the development and optimization of CE enantioselective methods, it is surprising that they haven’t been used more extensively. The relatively low number of applications of DoE in the development and optimization of CE chiral methods in the late 90′ and early 2000′ could be explained due to a lack of knowledge and access to specialized software. However, a clear tendency of applying multivariate approaches in CE chiral method development can be observed in the last ten years. It can be noted that DoE was implemented mostly in the development and optimization of CZE methods (as it is the most frequently used CE technique), and only in few NACE, CEC, MEKC and CE-MS methods.

Initial studies often employed a single design to establish the optimum analytical conditions, often in combination with an OFAT approach, while the current trends head towards the use of a screening design to identify significant experimental factors followed by an optimization design for fine-tuning the separation conditions. The latter strategy in most cases deserves to be preferred, being more reliable and effective. First, it enables to consider all the possible critical parameters, without the need to choose which parameters should be fixed or studied when carrying out optimization by RSM. Moreover, another advantage is the possibility of moving the experimental domain of some factors towards the zone leading to the best results, as the outcome of the screening phase. With this approach it is also possible to ward off some questionable procedures which nevertheless can be found in the literature, such as the introduction of further factors to be studied by OFAT after having completed the multivariate optimization.

Many variables can affect CE chiral separations, and this is critical in obtaining enantioresolution. In this regard, application of DoE is well suited for the development of enantioresolution methods because they provide an effective strategy for clarifying the impact of experimental conditions. Each design has its own set of features that can be used to meet specific requirements.

It is worth to be mentioned, that scouting experiments should precede the DoE strategy, in order to establish some basic experimental parameters, as well as the CE operative mode, and to assess the experimental domain. For example, the type of CS is typically selected in this early phase of method development.

In a recent review by Yu and Quirino [7], the CSs used in EKC have been grouped into: (a) macromolecular (including macrocyclic), (b) supramolecular, and (c) low molec-ular mass compounds, showing the vast choice of opportunities to face the enantiosepa-ration challenge. In the selection of the suitable CS, that is the first step towards in meth-od optimization, a rational approach should be desirable, and in the recent years a notable number of studies have involved molecular modelling and/or NMR to understand the chiral recognition mechanism of CSs. In some specific cases, the achieved information has allowed for the enantioselectivity of the CS towards a chiral analyte, to be predicted. Moreover, by means of the described approach, the classes of analytes that can be separated by a given CS could be predicted as well. Nevertheless, the choice of the suitable CS for each target, is usually still carried out by trial and error approach. Generally, a screening of different CD derivatives (neutral and negatively/positively charged) at several pH values (where the analyte is found under ionized and/or unionized form) is performed. The optimal CS is then selected based on resolution, analysis time, peak shape, or other quality characteristics of the separation system. Importantly, this strategy must be critically approached by the researcher which should deeply know all of the characteristics of the analyte (e.g., molecular mass, hydrophobicity, pKa value/s etc.) in order to restrict the number of potentially useful CSs for the considered purpose. However, sometimes the type of the CS can be introduced as qualitative factor in the screening design; this can be advantageous, because the enantioseparation power of the CS can be tuned by the other experimental conditions and in this way a complete and systematic overview of the candidate selectors can be obtained.

Usually in chiral CE applied DoE, the experimental variables are BGE concentration, BGE pH, CS concentration, temperature, voltage; while the responses are chiral resolution, analysis time and sometimes generated current, peak symmetry, and efficiency. Typically, a number of 4–7 factors is evaluated in the screening phase, while in the optimization step the study is limited to 3–4 factors. It is fundamental to introduce in the screening all the parameters that could influence the method, in order to investigate the effect of all of them. The significant factors for a selected response are determined in the screening step. The optimal conditions are determined by evaluating the effect of the selected significant factors in the optimization step.

The analytical responses are usually chiral resolution and analysis time (migration time of the second enantiomer), but sometimes other responses like peak shape, described by values of peak efficiency (number of theoretical plates) and peak symmetry (peak asymmetry factor, tailing factor) are also used. The choice of the responses is usually made on the basis of the critical analytical issues which are evidenced in the first phase of method development. Moreover, in many cases, additional quantitative responses as migration times or peak areas are included in the robustness testing, factors which were not followed during method optimization.

During the screening stage, the most significant factors are separated from those that are thought to influence at a lesser extent the analytical responses. The factors that do not provide useful information on the behavior of the responses must be eliminated in this stage, leaving just the most important ones to be examined in the optimization process. Among screening designs, FrFD, D-oD, and PBD are highly efficient choices as they require fewer experiments than FFD. PBD is particularly attractive because it requires few experiments, however it is used mainly in the validation process, to verify method robustness, because it supports only two levels for each experimental factor. FrFD represents the most frequently used solution in chiral CE methods screening phase, and over half of the articles listed in Table 1 use this kind of design in the screening step.

After the significant factors have been identified, their influence on the analytical responses must be studied in order to define the conditions leading to the desired response. This phase involves creating a mathematical model which depicts the response’s behavior as the levels of the factors change. In the optimization process, RSM has been widely used; RSM aims to convert experimental data into a polynomial equation that best explains the response’s behavior, allowing for trustworthy predictions. Several optimization designs have been used, including CCD, OAD, BBD, or DD. CCD and especially FCCD is the most employed design in optimization of chiral CE methods. The response surface is estimated based on the design findings, allowing for the determination of optimal factor conditions. The selection of the factors to be investigated and their levels is the initial step of the optimization screening; only two or three variables are usually further optimized, even if the optimization dealing with four factors can still be easily managed with a reasonable number of experiments. In terms of the number of levels, three up to seven can be considered, depending on the design that was used. In some studies, RSM was used without performing a screening design, while the factors involved were selected based on the results of preliminary analysis.

The experimental setting is then devised and carried out, and the responses are measured. Following that, a polynomial model that explains the relationship between the response and the factors is constructed. ANOVA significance and validity are assessed together with the parameters describing the quality of the model in terms of fitting and prediction. Only after that, the model is evaluated graphically and/or statistically. The best experimental conditions are identified and experimentally tested in the last phase of method optimization using a response surface design.

In general, for carrying out the multivariate optimization of CE methods the researchers should be adequately trained in order to avoid pitfalls in the overall strategy. As a matter of fact, anyone, having at disposal a DoE software, can plan an experimental design, run the experiments, and obtain plots by graphical analysis of effects or by RSM. However, many issues can arise in the different steps of DoE workflow from a superficial knowledge of multivariate techniques.

First of all, the researcher should be aware of the theoretical principles which lead to the choice of the proper matrices according to their characteristics. A screening matrix cannot be used for predictions or for drawing response surfaces; at the same time, an optimization design without a proper selection of the variables made by a previous screening phase could represent a waste of resources which could be better addressed.

Moreover, the researcher should carefully define the limits of the experimental domain for the factors, and in most cases preliminary experiments are necessary for this choice. In fact, it is important that all the planned experiments can be carried out in a safe zone, where technical constraints do not occur, and all the considered responses can be measured. The constraints can include, for instance, a too high value of generated current, or a too low value of EOF for which the analytes cannot be drawn to the detector in a reasonable time, or a too high value of CS concentration for which the BGE cannot be prepared in practice. In order to be able to calculate the coefficients of the model, a sufficient number of measured responses should be available; if this does not happen, the whole experimental design can fail and a new one should be planned.

Another possible shrewdness for avoiding subsequent issues in data treatment is to give a first critical look to the data before including the responses in the software for calculating the model. In this way the researcher can identify the presence of inconsistent data which can be derived from wrong calculation of the responses or from exchange of rows in the matrix or simply from typing errors. These responses should be corrected in order to avoid including wrong data; in fact, in some cases they could be evidenced as outliers by the software, but if this does not happen, they contribute to the calculation of the model and thus to misleading results.

Another point of paramount importance is that in the optimization phase the model performances should be checked by calculation of ANOVA and of the model quality parameters, and that these data should be reported in the developed method. This is a step which is sometimes neglected by the researchers, but it has to be underlined that if the model results of poor quality, it cannot be used for drawing response surfaces, and it should be discarded.

Finally, the latest trends in the multivariate development of CE enantioseparation methods regard the implementation of QbD principles. This science and risk-based approach consists in a well-defined framework where the key points are the use of risk assessment tools, the multivariate study of the effect of critical process parameters, articulated in a screening and optimization phase, leading to the identification of the MODR and finally to the definition of method control. The main novelty of this approach is that the optimum is no more represented by a point, but by a multidimensional zone where the quality of the analytical performances is assured with a selected probability. This strategy can be particularly useful when the aim of the method is the routine control of the enantiomeric purity of a drug administered as single enantiomer. By QbD, an in-depth understanding of the interactions and of the criticality of procedure parameters can be achieved. A higher confidence in the results is generated, with a wider knowledge and an outcome represented by more robust methods and by a greater regulatory flexibility, flexibility, since no revalidation of the method is required for changes within the MODR.

## Figures and Tables

**Table 1:** Chiral CE methods for the analysis of pharmaceuticals developed by using experimental design strategies.

Analytes	Sample	Experimental Design Type	Factors	Responses	Optimized Conditions	References
Clenbuterol	bulk substance	PBD	BGE pH, BGE concentration, CD concentration, methanol concentration, injection time	resolution, migration time, number of theoretical plates	0.1 M citric acid/0.2 M phosphate BGE, pH 4.0, 16 mM β-CD, 19 °C, 13 kV, 214 nm	[47]
Amlodipine	bulk substance	CCD	BGE pH, CD concentration, temperature	resolution, Kaiser peak separation function	50 mM phosphate BGE, pH 3.16, 18.2 mM α-CD, 17.2 °C, 25 kV, 214 nm	[48]
Dexfenfluramine-chiral purity (levofenfluramine)	bulk substance	PBD	BGE pH, CD concentration, methanol concentration, temperature, voltage	resolution, migration time, peak symmetry	100 mM phosphate BGE, pH 3.0, 10 mM DM-β-CD, 40% methanol, 20 °C, 25 kV, 214 nm	[50]
Amphetamines (amphetamine, methamphetamine, MDA, MDMA, MDEA)	bulk substance	CCD	BGE concentration, BGE pH, CD concentration, temperature, voltage	amphetamine resolution, total resolution, migration time, generated power	118 mM phosphate BGE, pH 3.5, 16 mM HP-β-CD, 28 °C, 25 kV, 200 nm	[51]
Epinephrine	bulk substance	FrFD CCD	BGE concentration, BGE pH, CD concentration, temperature, voltage BGE concentration, CD concentration, voltage	resolution, migration time	111 mM phosphate BGE, pH 7.0, 9 mM CM-β-CD, 20 °C, 18 kV, 206 nm	[52]
Imidazole derivatives (bifonazole, econazole, miconazole, sulconazole)	bulk substance	FrFD	BGE pH, acetonitrile concentration, CD concentration, temperature	resolution, migration time, number of theoretical plates	100 mM phosphate BGE, pH 4.70, 5.2% acetonitrile, 5.80 mM β-CD, 35 °C, 30 kV, 230 nm	[53]
Celiprolol	bulk substance, tablet, urine	FCCD	BGE concentration, BGE pH, CD concentration, temperature	resolution, migration time, current	52 mM acetate BGE, pH 4.0, 3 mM S-β-CD, 19.5 °C, 30 kV, 220 nm	[55]
Salbutamol	spiked urine	PBD DD	BGE concentration, BGE pH, CS concentration, methanol concentration, voltage BGE pH, CS concentration, voltage	resolution, migration time	30 mM Tris BGE, pH 5.3, 1.75% dermatan sulphate, 5% methanol, 15 °C, 24 kV, 220 nm	[59]
Atropine	ophthalmic solution, plant extract	CCD	BGE concentration, BGE pH, CD concentration	resolution, migration time, current	55 mM phosphate BGE, pH 7.0, 2.9 mM S-β-CD, 20 °C, 20 kV, 195 nm	[60]
Methadone	bulk substance	FFD FCCD	CD concentration, percentage of capillary filled with CD, gas nebulization pressure	resolution	CE-ESI-MS: 20 mM ammonium acetate BGE, pH 4.0, 18 mg/mL HP-β-CD/ 0.25 mg/mL CM-β-CD/ 0.4 mg/mL SBE-β-CD, 90/70/90% capillary-filled, nebulization pressure 2 psi	[110]
Lactic acid	body fluids (plasma, urine, amniotic and cerebrospinal fluid)	FFD FFD	BGE concentration, BGE pH, CS concentration BGE concentration, CS concentration	resolution	200 mM phosphate BGE, pH 6.0, 413 mM HP-β-CD, 20 °C,−20 kV, 200 nm	[63]
Propranolol	bulk substance	FCCD	BGE concentration, BGE pH, acetonitrile concentration	effective mobility, efficiency, symmetry, selectivity, relative mobility difference, resolution, difference in effective mobility	15 mM bistris-acetate, pH 6.5, 31.2 µM Cel7A, 17% (*v*/*v*) acetonitrile, 22 °C, 20 µA, 200 nm	[64]
2-[(4′-benzoyloxy-2′-hydroxy)phenyl-propionic acid]	bulk substance	CCD	CS concentration, BGE pH, temperature	resolution, migration time	50 mM Britton-Robinson BGE, pH 6.4, 7 mM vancomycin, 22 °C, 20 kV, 195 nm	[65]
Ofloxacin	bulk substance	FCCD	BGE concentration, BGE pH, CD concentration, temperature	resolution, migration time, peak area, current	50 mM phosphate BGE, pH 2.8, 4% (*w*/*v)* M-β-CD, 25 °C, 20 kV, 280 nm	[66]
Ketamine	injection solution	PBD CCD	BGE concentration, BGE pH, CD concentration, methanol concentration, voltage BGE pH, methanol concentration	resolution	50 mM phosphate BGE, pH 5.20, 2% (*w*/*v*) CM-β-CD, 30% methanol, 20 °C, 15 kV, 206 nm	[67]
Tamsulosin	bulk substance	BBD FCCD CCC	CD concentration, voltage, temperature	resolution, migration time	100 mM Tris BGE, pH 2.5, 0.15% (*w*/*v*) S-β-CD, 25 °C, 25 kV, 210 nm	[75]
Risperidone, 9-hydroxyrisperidone	bulk substance	CCD	CD_1_ concentration, CD_2_ concentration, BGE concentration	resolution, migration time, selectivity, number of theoretical plates	80 mM phosphate BGE, pH 2.5, 37 mM HP- β-CD, 3.7% S-α-CD, 25 °C, 20 kV, 270 nm	[76]
*S*-Citalopram--chiral purity (*R*-Citalopram)	bulk substance, tablets	FCCD	BGE concentration, CD_2_ concentration, voltage, temperature	resolution, migration time, current	20 mM phosphate BGE, pH 2.5, 0.5 mg/mL β-CD, 22 mg/mL S-α-CD, 28 °C, −20 kV, 205 nm	[77]
Propranolol, 4-hydroxypropranolol	bulk substance	BBD	BGE concentration, BGE pH, CD concentration, voltage	resolution, migration time	25 mM triethylamine/phosphate BGE, pH 9.0, 4% (*w*/*v*) CM-β-CD, 25 °C, 17 kV, 208 nm	[78]
Zopiclone	bulk substance, tablets	FFD	BGE concentration, BGE pH, CD concentration, voltage	resolution, migration time	60.2 mM phosphate BGE, pH 2.0, 1 M urea, 20 mM β-CD, 25 °C, 30 kV, 215 nm	[79]
Clopidogrel (chiral purity *R*-clopidogrel)	bulk substance	reduced CCD	BGE concentration, BGE pH, CD concentration, voltage	resolution, migration time	10 mM triethylamine–phosphoric acid BGE, pH 2.3, 5% S-β-CD, 20 °C, −12 kV, 195 nm	[80]
Dapoxetine	bulk substance	OAD	BGE concentration, BGE pH, CD concentration, methanol concentration, temperature, voltage	resolution	70 mM acetate BGE, pH 4.5, 3 mM M-γ-CD, 20% (*v*/*v*) methanol, 15 °C, 15 kV, 215 nm	[82]
Magnesium-*L*-aspartate (chiral purity *D*-aspartate)	bulk substance, tablets	FFD	BGE concentration, BGE pH, CD concentration	resolution, migration time	50 mM phosphate, pH 7, 18 mM HP-β-CD, 18% (*v*/*v*) DMSO, 20 °C, 30 kV, LIF detection	[87]
Asenapine	bulk substance	OAD	BGE concentration, BGE pH, CD concentration, methanol concentration, temperature, voltage	resolution	160 mM Tris-acetate BGE, pH 3.5, 7 mM β-CD, 20 °C, 15 kV, 214 nm	[89]
Pomalidomide	bulk substance	OAD	BGE concentration, BGE pH, temperature, voltage	resolution	50 mM Tris-acetate BGE, pH 6.5, 15 mM CM-β-CD, 20 °C, 15 kV, 215 nm	[90]
Lenalidomide (chiral purity *R*-lenalidomide)	bulk substance	FCCD	BGE concentration, temperature, voltage	resolution	30 mM phosphate BGE, pH 6.5, 30 mM SBE-β-CD, 10 °C, 12 kV, 210 nm	[91]
Vildagliptin (chiral purity *R*-vildagliptin)	bulk substance, tablets	OAD	BGE concentration, BGE pH, CD concentration, temperature, voltage, injection parameters	resolution	75 mM Tris-acetate BGE, pH 4.75, 20 mM SBE-α-CD, 15 °C, 25 kV, 40 mbar × 4 s hydrodynamic injection, 200 nm	[92]
Levosulpiride, (chiral purity *R*-sulpiride)	bulk substance, tablets	Asymmetric screening matrix DD	BGE concentration, BGE pH, type of neutral cyclodextrin, charged CD concentration, neutral CD concentration, voltage BGE pH, charged CD concentration, neutral CD concentration, voltage	resolution, migration time	5 mM Britton-Robinson BGE, pH 3.45, 10 mM S-β-CD, 34 mM M-β-CD, 16 °C, −14 kV, 214 nm	[88]
Ambrisentan (chiral purity *R*-ambrisentan)	bulk substance, coated tablets	Asymmetric screening matrix FCCD	BGE concentration, BGE pH, CD concentration, SDS concentration, temperature, voltage, capillary length BGE pH, CD concentration, voltage	resolution, migration time	capillary total length 64.5 cm, 100 mM borate BGE, pH 9.20, 100 mM SDS, 50 mM γ-CD, 22 °C, 30 kV, 200 nm	[112]
*S*-Ambrisentan (chiral purity *R*-ambrisentan)	bulk substance	FrFD FCCD	concentration, BGE pH, CD concentration, temperature, voltage BGE concentration, temperature, voltage	resolution, migration time	50 mM sodium acetate BGE, pH 4.0, 30 mM γ-CD, 25 °C, 25 kV, 200 nm	[93]
Montelukast (chiral purity *R*-trans-montelukast, *R*,*S*-cis-montelukast, *S*,*R*-cis-montelukast)	bulk substance, chewable tablets, oral granules	FFD	BGE concentration, voltage	resolution	20 mM borate BGE, 10 mM SDS, pH 9.0, 10 mM HTM-β-CD, 12 mM SBE-β-CD, 15 °C, 18 kV, 254 nm	[113]
Levomepromazine (chiral purity *R*-mepromazine)	bulk substance, injection solution	FrFD FCCD	BGE concentration, BGE pH, CD concentration, temperature, voltage BGE concentration, BGE pH, CD concentration	resolution, migration time, selectivity	100 mM citric acid BGE, pH 2.85, 3.6 mg/mL HP-γ-CD, 15 °C, 25 kV, 253 nm	[114]
*S*-Dapoxetine (chiral purity *R*-Dapoxetine)	bulk substance, tablets	FrFD FCCD	BGE concentra-tion, BGE pH, CD_1_-CD_2_ 1:1 mix concentration, temperature, voltage CD_1_ concentra-tion, CD_2_ concentration, voltage	resolution, migration time, peak symmetry, current	50 mM phosphate BGE, pH 6.3, 45 mg/mL S-γ-CD, 40.2 mg/mL DM-β-CD, 15 °C, 9 kV, 215 nm	[83]
Sitafloxacin, chiral impurities	bulk substance	FrFD FCCD	BGE concentration, BGE pH, CD concentration, Cu^2+^ concentration, *D*-Phe concentration, temperature, voltage BGE pH, CD concentration, Cu^2+^ concentration, *D*-Phe concentration	resolution, migration time	15 mM phosphate BGE, pH 4.5, 15 mM *D*-Phe, 20 mM CuSO_4_, 20 mM γ-CD, 25 °C, 15 kV, 297 nm	[94]
Fluoxetine	bulk substance, tablets	OAD	BGE concentration, BGE pH, CD concentration, temperature, voltage, injection parameters	resolution	50 mM phosphate BGE, pH 5.0, 10 mM HTM-β-CD, 15 °C, 20 kV, 50 mbar × 1 s hydrodynamic injection, 230 nm	[95]
Pregabalin	bulk substance, capsules	D-oD FCCD	BGE concentration, BGE pH, CD concentration, temperature, voltage CD concentration, temperature, voltage	resolution, migration time	100 mM phosphate BGE concentration, pH 2.5, 40 mg/mL HTM-β-CD, 25 °C, 15 kV, 220 nm	[96]
Dexmedetomidine (chiral purity *R*-medetomidine)	bulk substance, tablets	FrFD FCCD	BGE concentration, BGE pH, CD concentration, temperature, voltage CD concentration, temperature, voltage	selectivity, migration time, current	50 mM phosphate BGE, pH 6.5, 40 mg/mL S-β-CD, 17 °C, 10 kV, 200 nm	[97]
Dextrometorphan (chiral purity *R*-metorphan)	bulk substance, tablets	FrFD FCCD	BGE concentration, BGE pH, CD concentration, temperature, voltage CD concentration, voltage	resolution, migration time	30 mM phosphate BGE, pH 6.5, 16 mg/mL S-β-CD, 14 mg/mL M-α-CD, 20 °C, 20 kV, 200 nm	[98]
Cinacalcet (chiral purity *S*-cinacalcet)	bulk substance, tablets	BBD	BGE pH, CD concentration, methanol concentration, voltage	resolution, migration time	150 mM phosphate BGE, pH 2.7, 3.1 mM HP-γ-CD, 2% (*v*/*v*) methanol, 18 °C, 26 kV, 220 nm	[99]
Benzodiazepines (lorazepam, lormetazepam, oxazepam, temazepam)	bulk substance	FrFD	BGE pH, CD type, CD concentration, methanol concentration	resolution	20 mM borate BGE, pH 9.0, 5% HES-β-CD, 15% methanol, 15 °C, 20 kV, 230 nm	[100]
Lansoprazole, Rabeprazole	bulk substance, capsules	FrFD CCD	BGE concentration, BGE pH, CD_1_ concentration, temperature, voltage CD_1_ concentration, voltage, temperature	resolution, migration time	Lansoprazole: 25 mM phosphate BGE, pH 7.0, 10 mM SBE-β-CD, 20 mM γ-CD, 17 °C, 20 kV, 210 nm Rabeprazole: 25 mM phosphate BGE, pH 7.0, 15 mM SBE-β-CD, 30 mM γ-CD, 18 °C, 20 kV, 210 nm	[104]
Levodropropizine (chiral purity *R*-dropropizine)	bulk substance, pharmaceutical drops	FFD FCCD	CD concentration, temperature, voltage, 2-propanol concentration CD concentration, temperature	separation factors, migration time, current	25 mM phosphate BGE, pH 7.0, 23.5 mg/mL S-β-CD, 10% (*v*/*v*) 2-propanol, 16.3 °C, 16.5 kV, 200 nm	[106]
Levomepromazine (chiral purity *R*-mepromazine)	bulk substance, tablets	FrFD FCCD	BGE concentration, CD concentration, temperature, voltage BGE concentration, CD concentration, temperature	resolution, migration time, current	750 mM acetic acid, 55 mM ammonium acetate in methanol BGE, 27.5 mg/mL HDMS-β-CD, 15 °C, 22 kV, 250 nm	[120]
Tramadol	bulk substance, capsules	FCCD	BGE pH, CD concentration, temperature	resolution, migration time	25 mM tetraborate BGE, pH 11.0, 5 mM CM-β-CD, 15 °C, 17.5 kV, 210 nm	[105]
Amlodipine	bulk substance, tablets	OAD	BGE concentration, BGE pH, CD concentration, temperature, voltage, injection parameters	resolution	25 mM phosphate BGE, pH 9.0, 15 mM CM-β-CD, 15 °C, 25 kV, 30 mbar × 1 s hydrodynamic injection, 230 nm	[107]
Citalopram	bulk substance, tablets	FrFD FCCD	BGE concentration, BGE pH, CD concentration, temperature, voltage, injection pressure CD concentration, temperature, voltage	resolution, migration time	25 mM phosphate BGE, pH 7.0, 3 mM CM-β-CD, 17.5 °C, 15 kV, 50 mbar injection pressure, 230 nm	[108]
Venlafaxine	bulk substance, capsules	FCCD	CD concentration, temperature, voltage	resolution, migration time	25 mM phosphate BGE, pH 2.5, 10 mM CM-β-CD, 15 °C, 25 kV, 230 nm	[109]

## Abbreviations

ANOVA	Analysis of variance
AQbD	Analytical Quality by Design
ASD	Asymmetric screening design
BBD	Box-Behnken design
BGE	background electrolyte
CCC	Central composite circumscribed design
CCD	Central composite design
CD	cyclodextrin
CE	capillary electrophoresis
CEC	capillary electrochromatography
CE-MS	capillary electrophoresis—mass spectrometry
CE-ESI-MS	capillary electrophoresis coupled with electrospray ionization mass spectrometry
CMA	Critical Method Attribute
CMP	Critical Method Parameter
CM-β-CD	carboxymethyl-β-cyclodextrin
CPP	critical process parameter
CQA	critical quality attributes
CS	chiral selector
CSP	chiral stationary phase
CZE	capillary zone electrophoresis
D-oD	D-optimal design
D-Phe	D-Phenylalanine
DoE	Design of Experiments
DD	Doehlert design
DM-β-CD	dimethyl-β-cyclodextrin
DS	Design Space
EMA	European Medicines Agency
EOF	electroosmotic flow
FDA	Food and Drug Administration
FCCD	Face centered central composite design
FFD	Full factorial design
FLEC	l-(9-fluorenyl)ethyl chloroformate
FMOC	9-fluorenylmethyl chloroformate
FrFD	Fractional factorial design
HDAS-β-CD	heptakis(2,3-di-O-acetyl-6-O-sulfo)-β-cyclodextrin
HDM-β-CD	heptakis-2,6-di-O-methyl-β-cyclodextrin
HDMS-β-CD	heptakis-(2,3-di-O-methyl-6-O-sulfo)-β-cyclodextrin
HES-β-CD	heptakis-6-sulfato-β-CD
HMAS-β-CD	heptakis(2-O-methyl-3-O-acetyl-6-O-sulfo)-β-cyclodextrin
HPLC	high performance liquid chromatography
HP-β-CD	hydroxypropyl-β-cyclodextrin
HP-γ-CD	hydroxypropyl-γ-cyclodextrin
HS-β-CD	highly sulphated-β-cyclodextrin
HTM-β-CD	heptakis (2,3,6-tri-O-methyl)-β-cyclodextrin
HSA	human serum albumin
ICH	International Council for Harmonisation
LIF	laser induced fluorescence
LOD	limit of detection
LOQ	limit of quantification
M-α-CD	methyl-α-cyclodextrin
M-β-CD	methyl-β-cyclodextrin
M-γ-CD	randomly methylated γ-cyclodextrin
MDA	3,4-methylenedioxyamphetamine
MDEA	3,4-methylenedioxyethylamphetamine
MDMA	3,4-methylenedioxymethamphetamine
MEKC	micellar electrokinetic chromatography
MODR	Method operable design region
NACE	nonaqueous capillary electrophoresis
OAD	Orthogonal array design
ODMS-γ-CD	octakis(2,3-di-O-methyl-6-O-sulfo)-γ-cyclodextrin
OFAT	one factor at a time
PBD	Plackett-Burman design
PEG	polyethylene glycol
PO	polar organic
QbD	Quality by Design
RP	reverse phase
RSM	Response Surface Methodology
S-β-CD	sulfated-β-CD
S-γ-CD	sulfated-γ-CD
SBE-β-CD	sulfobutylether-β-cyclodextrin
SDS	sodium dodecyl sulfate
S/N	signal-to-noise
SSD	Symmetric screening design
SNRI	serotonin and norepinephrine reuptake inhibitor
SSRI	selective serotonin reuptake inhibitor
